# Exposomic Assessment of Endocrine-Disrupting Chemicals Using Hair: A Cross-Matrix Comparison with Serum and Urine by LC-HRMS and Metabolite Profiling

**DOI:** 10.1021/acs.est.6c05029

**Published:** 2026-09-11

**Authors:** Ana González-Ruiz, Adrià Sunyer-Caldú, Rodrigo Braz Carneiro, Esteban Restrepo-Montes, Olivia Ballester-Miró, Alejandro Lobato-Berezo, Ramón M. Pujol, Montse Marquès, Rubén Gil-Solsona, Pablo Gago-Ferrero

**Affiliations:** † Laboratory of Toxicology and Environmental Health, School of Medicine, TecnATox, Universitat Rovira i Virgili (URV), Sant Llorenç 21, 43201 Reus, Spain; ‡ 537431 Institut d’Investigació Sanitària Pere Virgili (IISPV), Doctor Mallafré Guasch 4, 43005 Tarragona, Spain; § Department of Environmental Science (ACES, Exposure & Effects), Science for Life Laboratory, 7675 Stockholm University, Stockholm 106 91, Sweden; ∥ São Carlos School of Engineering, 28133 University of São Paulo (EESC-USP), João Dagnone Ave 1100, São Carlos 13563-120, Brazil; ⊥ Department of Environmental Chemistry, 203229 Institute of Environmental Assessment and Water Research (IDAEA), Spanish Council of Scientific Research (CSIC), Jordi Girona 18-26, 08034 Barcelona, Spain; # Hospital Universitari Mútua Terrassa, Plaça Dr. Robert 5, 08221 Terrassa, Spain; ∇ 16548 Institut Hospital del Mar, Passeig Marítim 25-29, 08003 Barcelona, Spain

**Keywords:** EDCs, human biomonitoring, high-resolution mass spectrometry, hair analysis, environmental exposure, exposomics

## Abstract

Endocrine-disrupting chemicals (EDCs) are increasingly implicated in chronic diseases, yet their biomonitoring in human studies still relies mainly on urine and serum, which capture only short-term exposure to these fast-elimination compounds. Here, we evaluated hair as a complementary and long-term biomonitoring matrix for EDC mixtures by integrating it into an LC-HRMS exposomics workflow together with paired serum and urine samples. We developed and validated a method targeting 44 endocrine disruptors from five chemical families (benzophenones, phthalates, parabens, bisphenols, and organophosphate esters), combining quantitative analysis with suspect screening and semi-quantification of phase II metabolites. Applied to samples from 20 postmenopausal women, the hair-based approach provided the broadest coverage and highest concentrations (31/44 compounds), up to about three orders of magnitude higher than in urine or serum, consistent with distinct accumulation mechanisms and temporal integration windows. In hair, EDCs were present in unconjugated form (>99%), whereas urine was dominated by conjugated metabolites and serum showed limited detection. Overall, these findings demonstrate that integrating hair into multimatrix exposomics designs substantially expands both the chemical and temporal resolution of human exposure assessment.

## Graphical abstract

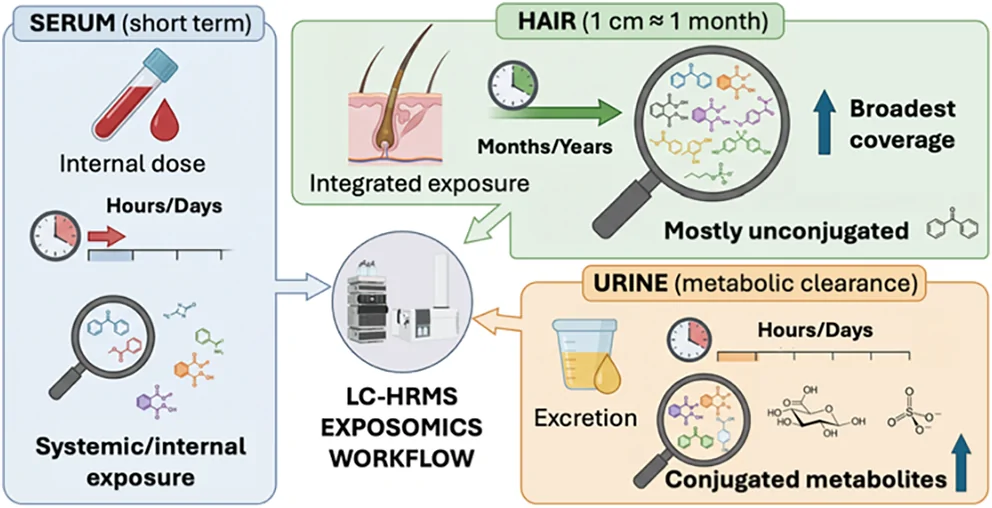

## 1 Introduction

Humans are constantly exposed to a myriad of chemicals, many of which are potentially harmful, and whose cumulative effects remain poorly characterized.^1^ The intricate processes behind disease etiology are generally influenced by a combination of individual genetic predispositions and environmental factors. However, recent evidence indicates that most chronic diseases are linked to environmental exposures,^2,3^ making the quantitative measurement of chemical exposures a cornerstone of modern exposomics. Accurately assessing long-term exposure to these chemicals remains a major challenge in this field. Sampled concentrations should reflect the levels to which individuals are exposed over time rather than transient or isolated peaks, an issue that has become increasingly addressable through recent advances in analytical methodologies and computational approaches.

Among the vast universe of chemicals to which humans are frequently exposed, endocrine disruptors (EDCs) are particularly concerning due to their well-documented adverse health effects, as they interfere with the normal functioning of the endocrine system.^4^ EDCs encompass chemicals such as parabens, benzophenones, bisphenols, phthalates, or flame retardants. These chemicals are commonly included in consumer products, including cosmetics, food packaging, household cleaning products, and pharmaceutical excipients, and their continuous release into indoor and personal environments leads to ubiquitous human exposure.^5–7^ Exposure to these chemicals has been linked to various health effects, including reproductive and developmental disorders,^8,9^ obesity and diabetes,^10,11^ neurodevelopmental conditions,^12,13^ and cancer.^14,15^ Yet most biomonitoring studies remain restricted to single matrices and short exposure windows, providing only a partial picture of an individual’s chemical burden. Integrating matrices with complementary exposure windows, together with broad chemical and metabolite coverage, is essential to more comprehensively capture real-world exposures.

Human biomonitoring of EDCs still relies largely on urine and serum, which provide valuable but short-term exposure snapshots due to the rapid metabolism and excretion of most of these compounds.^16^ A recent exposomics study on blood plasma samples revealed significant variability and limited longitudinal stability in individual chemical exposures, underscoring the need for more frequent measurements and complementary biospecimens to accurately capture chronic exposure profiles.^17^ Hair offers a promising alternative in this context. As a keratinized matrix that grows continuously, it reflects exposure over extended periods and can accumulate a wide range of chemicals,^18^ offering a biologically integrated record of exposure history over weeks to months (typically ∼1 cm of growth per month), although incorporation efficiencies depend on compound-specific physicochemical properties. Fäys et al. demonstrated that pollutant levels in hair exhibit lower temporal variability compared to urine, reporting consistently higher intraclass correlation coefficients (ICC) for hair biomarkers.^19^ Additionally, recent work in hair biomonitoring has directly assessed organophosphorus flame-retardant (OPFRs) levels in hair with those in urine and blood, reporting substantially higher detection frequencies and concentrations in hair, alongside weak correlations across matrices.^20^ These findings exemplify the lower variability and higher temporal stability of hair, supporting its suitability for chronic exposure assessment. Hair sampling also offers practical advantages: it is non-invasive, easy to collect, and requires minimal storage resources, making it a convenient option for large-scale epidemiological studies.^18^

Research on EDCs in hair is still limited compared to other contaminants, such as pesticides.^21–27^ Bisphenols, such as bisphenol A (BPA) and bisphenol S (BPS), are the most commonly investigated EDCs in hair, with studies reporting their widespread presence from low ng/g to μg/g.^19,28–30^ Phthalates, organophosphate esters (OPEs), and, to a lesser extent, parabens, have also been detected in hair in several cohorts, though they are generally less studied.^30–38^ Benzophenones, used as UV filters, have been analyzed in a few studies, showing varying detection rates and concentrations in the low-ng/g range.^35,39^

Despite growing evidence of EDCs in hair, comparative data in paired biospecimens from the same individuals remain scarce, limiting our ability to evaluate how hair complements conventional matrices such as serum and urine in exposure assessment. Existing studies have primarily focused on a limited number of compounds or chemical families, and systematic cross-matrix evaluations remain uncommon. Fäys et al. and Li et al. investigated correlations between phthalate biomarkers in hair and urine,^19,40^ while Kucharska et al. and, more recently, Lallmahomed et al. explored selected flame retardants across hair, urine, and serum.^20,41^

To address these gaps, we developed and validated a target LC-HRMS-based method for the quantification of 44 EDCs spanning five chemical families (bisphenols, phthalates, parabens, benzophenones, and organophosphate esters) and applied it to paired hair, serum, and urine samples from the same individuals. In parallel, a complementary suspect screening workflow was implemented to identify additional phase II metabolites (glucuronides and sulfates), which were subsequently semi-quantified using an ionization efficiency-based model. We applied this combined workflow to 20 postmenopausal women to characterize real-world interindividual variability in multimatrix EDC profiles. This represents one of the first systematic comparisons of EDCs across three matrices in the same participants, enabling direct evaluation of hair’s complementarity to conventional biomonitoring approaches. Our primary objective is to demonstrate how multimatrix exposomics designs incorporating hair can substantially expand both the chemical coverage and temporal resolution of human exposure assessment.

## 2 Materials and Methods

### 2.1 Study Cohort

Paired hair, urine, and serum samples were collected from 20 postmenopausal women recruited at the Hospital del Mar (Barcelona, Spain) between October and December 2023. Ten participants had a confirmed clinical diagnosis of frontal fibrosing alopecia (FFA), and ten age-matched women without alopecia served as controls. Samples were used to test the analytical workflow and evaluate the intermatrix comparability of endocrine disruptor profiles across paired biospecimens. All participants provided an information sheet and written informed consent. The study was approved by the Research Ethics Committee for Drugs of the Parc de Salut Mar (CEIm-PSMAR, No. 2022/10348).

### 2.2 Sample Collection

For each participant, a bundle of hair strands was cut from the frontal area of the head, as close to the scalp as possible, and wrapped in aluminum foil. For participants with frontal fibrosing alopecia, samples were collected from areas with sufficient remaining hair density. Although the occipital region is commonly recommended in hair biomonitoring studies, regional differences in hair composition and analyte concentrations across the scalp have been reported; therefore, a single standardized sampling site was used for all participants to minimize within-study variability.^42,43^ Information on hair treatments (e.g., dyeing, bleaching, or straightening) and hair washing frequency was not available for this cohort. The foil was marked to indicate the orientation of the strands, distinguishing the proximal (near the root) and distal ends. Each sample was then placed in an individual envelope and stored at room temperature. Only the first 6 cm of the proximal part of the strands were used for analysis, corresponding to approximately the previous six months of exposure, assuming an average hair growth rate of 1 cm per month, although individual growth rates may vary.^44^ For urine collection, participants provided a 10 mL sample of their first-morning urine in a polypropylene container at their next visit. Blood samples were collected by trained nurses at Hospital del Mar using 10 mL Vacutainer tubes containing K2EDTA. Both urine and blood samples were stored at −80°C until analysis.

### 2.3 Chemicals, Reagents, and Analyte Selection

A total of 44 EDCs were selected, including bisphenols, phthalates, parabens, benzophenones, and flame retardants. The selection was based on a prioritization that considered (i) widespread human exposure documented in biomonitoring studies, based on production and consumption data from publicly available chemical databases and regulatory reports and on detection frequencies reported in previous works,^32,38,40,45,46^ (ii) relevance as established or suspected endocrine disruptors according to current regulatory and scientific evidence,^47^ and (iii) analytical compatibility with an LC-HRMS workflow applicable across hair, serum, and urine, including favorable physicochemical properties for bioaccumulation (log *P* range: 0.8–7.4). Several emerging bisphenol analogues (included in Table S1) were initially included in the analytical method, but were not detected in any sample and are therefore not discussed further. Additional information about analytes and internal standards is provided in Tables S1 and S2. Although several isotopically labeled standards structurally related to target analytes were included, labeled analogues were not available for all chemicals. Consequently, internal standards were selected to cover a broad range of physicochemical properties and chromatographic regions. Details on the preparation of standards and sources of materials can be found in Section S1.

### 2.4 Sample Treatment

Extraction protocols for serum and urine were based on previously developed methods.^48,49^ Briefly, 150 μL of serum were deproteinized with acetonitrile, centrifuged at 12,500 rpm for 10 min, and the supernatant was stored for injection. For urine samples, an aliquot of 500 μL was centrifuged (3500 rpm, 5 min) and filtered using Captiva cartridges. Creatinine levels for sample normalization were quantified using a COBAS Mira biochemical analyzer from Roche Diagnostics (Rotkreuz, Switzerland).

The extraction protocol for hair samples was adapted from Di Corcia et al.,^50^ using solvent-based washing steps similar to those employed in previous hair biomonitoring studies to minimize surface contamination prior to chemical analysis.^51–53^ Initially, hair samples were cleaned with 2 mL of dichloromethane and 2 mL of methanol, vortexed twice for 3 min with each solvent using a Heidolph Reax Top vortex mixer (Heidolph Instruments, Germany) at an intermediate speed setting (approximately 1200–1500 rpm), and dried at room temperature for 2 h. Subsequently, the samples were ground by bead beating in 2 mL screw-cap polypropylene microtubes containing ten 3 mm stainless steel beads using a FastPrep-24 5G Tissuelyzer (MP Biomedicals, Irvine, USA). Approximately 50 mg of pulverized hair (dry weight, accurately weighed) were extracted with 1 mL of methanol and incubated at 45 °C for 20 h in an orbital shaker. This methanol-based extraction approach is consistent with previously reported multiclass hair biomonitoring methods for EDCs.^35,38,54^ The extract was then transferred to an HPLC vial, evaporated to dryness under an N_2_ stream, reconstituted with 50 μL of H_2_O/MeOH (50:50 v/v), and stored until injection.

All samples were spiked before extraction with clothianidin-d3 as a surrogate standard to monitor extraction performance at a concentration of 50 ng/mL (in vial). Additionally, a mixture of 21 internal standards (see Table S2) was added prior to injection to account for instrumental response variations. Samples were stored at −80°C until analysis. Hair concentrations were normalized to sample weight, and urine concentrations were adjusted for creatinine levels.

### 2.5 Method Validation

Method validation parameters for urine and serum analysis can be found elsewhere.^48,49^ The hair extraction protocol was validated in terms of accuracy, precision, matrix effects, sensitivity, and linearity for 44 EDCs. Accuracy and precision were evaluated by spiking EDC native standards at 50 ng/mL into hair samples before extraction and analysis in triplicate. Recoveries were calculated from triplicate measurements by comparing the measured concentrations after extraction to the spiked concentration, while precision was assessed by calculating the standard deviation of the triplicate injections. Matrix effects were determined by comparing the slope of matrix-matched calibration curves, prepared by spiking native standards into pooled hair extracts after the extraction step, to the slope of solvent-based calibration curves. As standards were added postextraction, the resulting values reflect ionization-related phenomena independent of extraction recovery. For chemicals lacking sufficient calibration points (a minimum of 3) in hair due to their high concentration in the samples, the matrix effect was calculated by comparing the intensity at 50 ng/g on the matrix-matched calibration curve to the intensity on the solvent calibration curve, expressed as a percentage. Linearity was evaluated using matrix-matched calibration curves with 10 points ranging from 0.05 ng/g to 250 ng/g. The linear range was defined as the interval between the limit of quantification (LOQ) and the maximum point of the matrix-matched calibration curve within the acceptable range of linearity up to 250 ng/g for all chemicals. For real sample analysis, chemicals detected outside the linear range were quantified after diluting both the samples and calibration curves with MeOH/H_2_O (50:50) by factors of 10 and 100, followed by reinjection to ensure accurate results. The sensitivity was estimated using the LOQs. For chemicals absent in the pooled sample matrix, the LOQ was set as the lowest detectable concentration of the calibration curve. For chemicals already present in the pooled matrix or detected in procedural blanks, the LOQ was estimated as the average concentration measured in procedural blank samples, quantified using the solvent calibration curve, and corrected for matrix effects.

### 2.6 Quality Assurance and Quality Control

To assess potential contamination during sampling and extraction, we prepared field blanks for each matrix. Urine blanks consisted of Milli-Q water stored in polypropylene containers under the same conditions and for the same duration as those of the samples prior to analysis. Blood blanks were prepared by following the same collection procedure using K_2_EDTA tubes filled with Milli-Q water. Hair blanks consisted of precleaned aluminum foil exposed and processed in parallel to capture potential environmental contamination. To monitor and prevent contamination during sample preparation and instrumental analysis, procedural blanks (*n* = 4) were processed using the same protocol with Milli-Q water. Samples were extracted and analyzed in a single batch. Solvent blanks and quality control injections containing a mix of ISs at 50 ng/mL were included throughout the sequence to control potential carryover in the instrument and ensure signal stability and repeatability. More details are provided in Section S2.

### 2.7 LC-HRMS Acquisition

Analyte separation was performed with a Bruker Elute OLE chromatography system, equipped with an Intensity Solo analytical column (2.1 mm × 100 mm, 1.8 μm) from Bruker and a guard column Cortecs C18 (2.1 mm × 5 mm, 1.7 μm) from Waters (Milford, USA). Gradient time was 20.5 min, with a flow rate of 0.3 mL/min and an injection volume of 10 μL. The chromatographic system was coupled to a QTOF Impact II (Bruker, Bremen, Germany) mass spectrometer equipped with a heated electrospray ionization source (HESI) operating in both positive and negative ionization modes. Data Independent Acquisition mode (DIA) was employed with broadband collision-induced dissociation (bbCID) at a rate of 3 scans per second. Details on mobile phases, elution gradient conditions, and additional chromatographic and instrument parameters are provided in Section S3 and Tables S3 and S4.

### 2.8 Target Analysis, Suspect Screening of Metabolites, and Semi-Quantification

A targeted analysis of 44 EDC exposure biomarkers (including 12 phase I metabolites) was performed, for which accurate retention times and clear mass spectra were obtained under the described experimental conditions. For quantification, concentrations measured in the procedural blanks, along with three times their standard deviation, were subtracted from the concentrations in the samples for each chemical (see also Section 2.5). TASQ 2022.1.1 and DataAnalysis 5.3 (Bruker Daltonics, Bremen, Germany) were used for the data processing. Quantification was based on the parent peak in the extracted ion chromatogram, while compound identification and confirmation were supported by MS2 spectra and retention time alignment.

Suspect screening was used to screen for additional metabolites of EDCs present in the samples. DIA acquisition using bbCID was selected to ensure comprehensive MS2 coverage across a broad range of target and suspect compounds. Unlike DDA approaches, bbCID acquires fragmentation information for all detectable ions, improving the detection of low-abundance metabolites. Metabolic pathways were determined based on toxicokinetic literature,^55–59^ focusing on both phase I and phase II biotransformations. Metabolites from glucuronidation were identified by a mass increase of 176.0317, corresponding to the addition of C_6_H_10_O_7_ (glucuronic acid) and the loss of H_2_O, while sulfation products showed a mass increase of 79.9569, corresponding to the addition of SO_3_. These transformations increase the polarity of phase II metabolites, resulting in earlier retention times in the chromatogram compared to their parent compounds. Furthermore, structural similarities between parent compounds and their metabolites were reflected in shared mass fragments in the mass spectra. Candidate metabolites were considered annotated when they fulfilled the following criteria: (i) detection of an MS1 peak with accurate mass within ±0.005 Da of the theoretical mass; (ii) retention time behaviour consistent with the expected polarity shift relative to the parent compound; (iii) isotopic pattern agreement with the proposed molecular formula; and (iv) the presence of at least one diagnostic MS2 fragment ion characteristic of the proposed conjugated structure. Based on these criteria, metabolite annotations were assigned to Schymanski confidence level 2b (probable structure by diagnostic evidence), as authentic reference standards were not available for confirmation.^60^ Ionization mode, *m*/*z*, main fragments, and retention times of the detected metabolites and their corresponding parent compounds are provided in Table S5.

Semi-quantification of metabolites was performed using an ionization efficiency-based approach, applying a quantitative structure–ionization relationship (QSIR) model to predict ionization efficiency based on molecular descriptors specific to each chemical. Details on the development and validation of the QSIR model can be found elsewhere.^49^ This approach constructs a chemical space using calibration curves established within the matrix, reducing quantification errors commonly associated with solvent-based calibrations by accounting for both matrix effects and ionization efficiency, thereby improving the reliability of the results.^61^ In matrices where the total amount of a given chemical derives from both the parent compound and its phase II metabolite, the total concentration (ng/mL or ng/g) was calculated by normalizing the metabolite concentration to the molecular weight of the parent compound, effectively removing the contribution of the glucuronide or sulfate moiety.

### 2.9 Statistical Analysis

Statistical analyses were conducted using R programming language, version 4.5.0,^62^ using packages tidyverse, data.table, ggbreak, VennDiagram, grid, gridextra, and readxl.^63–68^ Concentration data were summarized using medians and interquartile ranges (IQR), given the non-normal distribution of the data. Values below the LOQ were treated as non-detects and excluded from descriptive statistics, graphical representations, and correlation analyses. Imputation methods were deliberately avoided because they may introduce systematic bias, particularly when detection frequencies are low, consistent with recommendations for handling left-censored data in biomonitoring studies.^69^ Spearman correlation analyses were performed to explore relationships among matrices and between hair-to-serum ratios and physicochemical properties. 95% confidence intervals were calculated using Fisher’s *z*-transformation and, as these analyses were considered exploratory, no multiple-testing corrections were applied. Statistical significance was established at *p* < 0.05.

## 3 Results

### 3.1 Methods Validation

The analytical method was successfully validated in hair samples for the detection of 44 EDCs, in terms of LOQs, precision, matrix effects, and extraction recovery rates (Table S6). LOQs were calculated for each chemical (Figure 1A), ranging from 0.05 ng/g to 67 ng/g (median = 0.4 ng/g), aligning with previous literature values for hair matrices.^70^ Most chemicals (63%) showed LOQs of less than 1 ng/g. Bisphenol F and triphenyl phosphate exhibited the highest LOQs (20 ng/g and 67 ng/g, respectively). However, the factors underlying these elevated values differ between compounds. For triphenyl phosphate, the higher LOQ was mainly attributable to contamination observed in procedural blanks, reflecting the ubiquitous presence of this compound in laboratory materials and solvents. In contrast, the elevated LOQ for bisphenol F was primarily related to analytical sensitivity limitations in hair, as acceptable quantitative performance could only be achieved from the lowest calibration level of 10 ng/g onward, despite the absence of blank contamination. More selective extractions or stricter blank controls could improve sensitivity for these specific analytes. However, our extraction strategy was intentionally designed to prioritize chemical coverage across families with diverse polarity and volatility, which is essential for multifamily suspect screening workflows. Such trade-offs between analytical performance for individual compounds and broad chemical coverage are inherent to exposomics-oriented HRMS workflows and have been previously reported in other broad-scope methodologies.^71^

**Figure 1:**
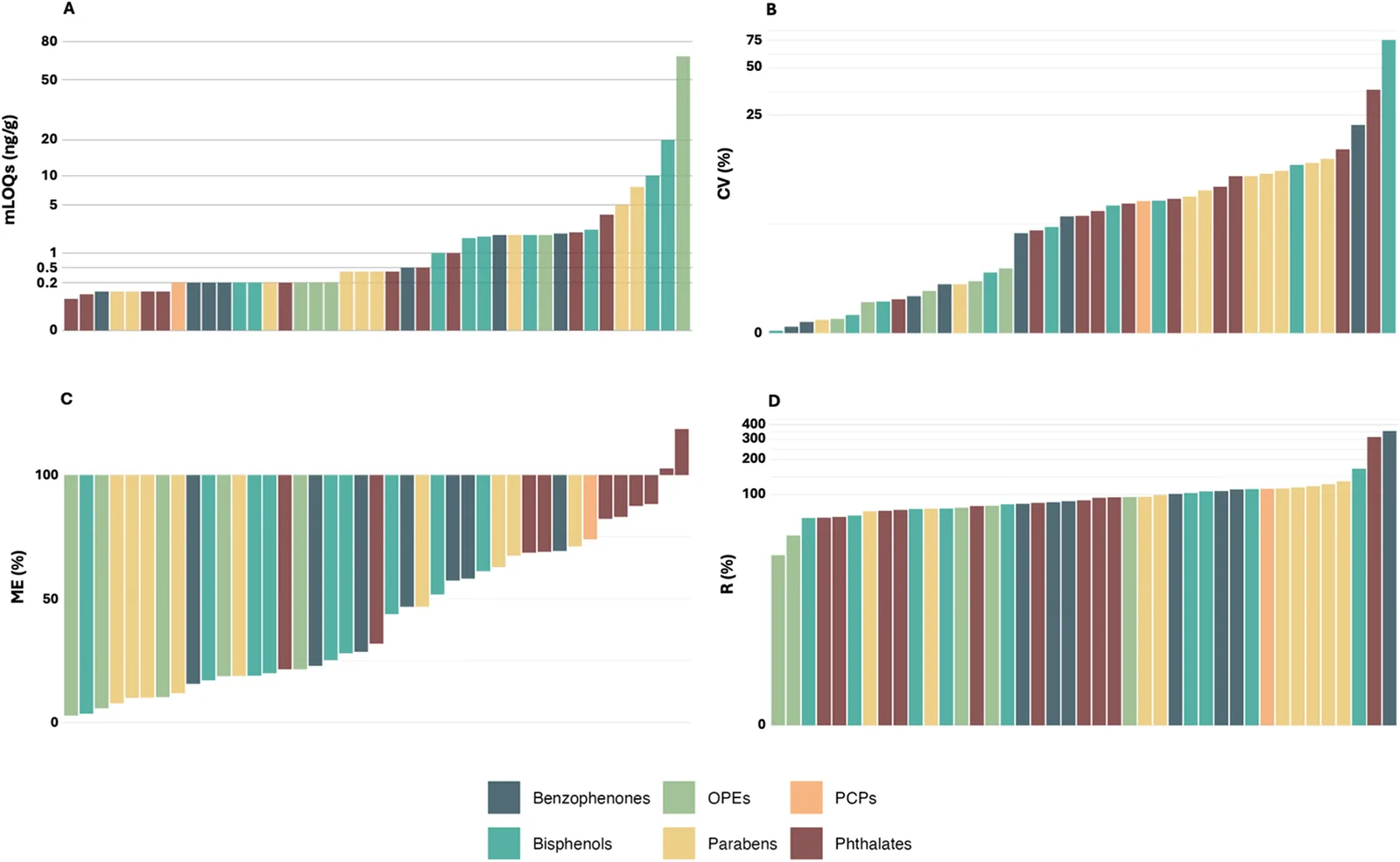
Validation parameters for the extraction method of 44 EDCs in hair samples colored by chemical families. (A) Method quantification limits for each compound (ng/g), (B) precision of the method, represented by coefficients of variation (CV) for each chemical, (C) matrix effect measurements, and (D) recovery rates across all chemicals (%). Each bar in the graph represents a different analyte. PCPs: Personal Care Products; mLOQ: Method Limit of Quantification; ME: Matrix Effect; R: Recoveries; CV: Coefficient of Variation.

All chemicals demonstrated good precision, with coefficients of variation (CVs) between 0.07% and 36.2% (Figure 1B), except for bisphenol M, which exhibited a CV value of 75.6% (not detected in any sample). As illustrated in Figure 1C, matrix effects predominantly resulted in signal suppression, with 33 chemicals exhibiting matrix effect values below 70%, likely due to the complexity of this matrix, while mono 2-ethyl-5-hydroxyhexyl phthalate (102.6%) and mono 2-ethyl-5-oxohexyl phthalate (118.6%) displayed signal enhancement. The analytes most affected by matrix effect were triethyl phosphate, bisphenol P, triphenyl phosphate, and isopropylparaben. Despite this limitation, accurate quantification was achieved using matrix-matched calibration curves, which effectively corrected for signal suppression. Additionally, internal standards from the same chemical families were used to further improve quantification reliability. Compound-specific recovery correction factors were also applied during quantification. Recoveries ranged between 50% and 130% for 78% of the analytes, indicating efficient extraction (Figure 1D). No additional normalization based on the response of the surrogate internal standard (clothianidin-*d*_3_) was performed, as its variability across the sample set was low (CV ∼15%). Tri-*o*-tolyl phosphate, mono 5-carboxy-2-ethylpentyl phthalate, bisphenol M, and triphenyl phosphate exhibited recoveries above this range. However, tri-o-tolyl phosphate and bisphenol M were not detected in any sample and therefore did not affect the reported results. Mono 5-carboxy-2-ethylpentyl phthalate was identified as an outlier among the 44 analytes, likely due to analytical overestimation or interferences, but it was also not detected in any sample. In contrast, triphenyl phosphate was detected in the samples and exhibited recovery values outside the acceptable range; therefore, concentrations were corrected based on its experimentally determined recovery. Benzophenone-4 was present at high concentrations in the pooled hair matrix (>200 ng/g), preventing accurate recovery assessment via standard spiking experiments. Thus, recovery was instead evaluated using a different hair matrix free of this analyte, from which matrix-matched calibration curves were prepared to ensure accurate quantification. Overall, the method provided consistent detection of a broad range of targeted EDCs in real hair samples, confirming its robustness for low-ng/g quantification within an exposomic framework.

Linearity was generally good, with *r* ^2^ values ranging from 0.9917 to 0.9999 for 75% of chemicals, with minor deviations observed for isobutyl paraben, butyl paraben, ethyl paraben, and propyl paraben, with *r* ^2^ values ranging from 0.9707 to 0.9866. Because of an insufficient number of data points in the matrix calibration curve, bisphenol S, triphenyl phosphate, and tris­(1-chloro-2-propyl) phosphate required the use of a solvent-based calibration curve and matrix effect corrections; after these adjustments, the chemicals exhibited good linearity with *r* ^2^ values ranging from 0.9945 to 0.9990. Further details on these validation parameters are provided in Table S6.

### 3.2 Distribution of Endocrine Disruptors across Biological Matrices

Total EDCs concentrations included both free forms (target analysis) and conjugated forms (suspect screening), normalized as described in Section 2.8 to allow direct comparison across matrices. This approach integrated all detected molecular forms into unified exposure metrics, ensuring comparability among matrices with distinct metabolic characteristics. Of the 44 EDCs analyzed, 33 were detected in at least one of the biological matrices. All conjugated forms corresponded to chemicals also detected in their free form in at least one biospecimen, although in some cases (notably urine), the conjugated species represented 100% of the total signal (see Section 3.3). Hair samples exhibited the highest chemical coverage, with 31 detected chemicals (70% of total compounds), followed by urine with 25 (57%) and serum with 14 (32%) (Figure 2). This trend clearly indicates that hair captures the broadest chemical coverage among the matrices. This observation is consistent with Hardy et al., who reported 24 chemicals found in over 40% of individuals using hair samples, compared to only 12 in urine.^26^ Quantitatively, 60% of the chemicals detected in hair overlapped with those in urine and 45% with serum, confirming substantial but incomplete overlap among matrices and emphasizing their complementarity.

**Figure 2:**
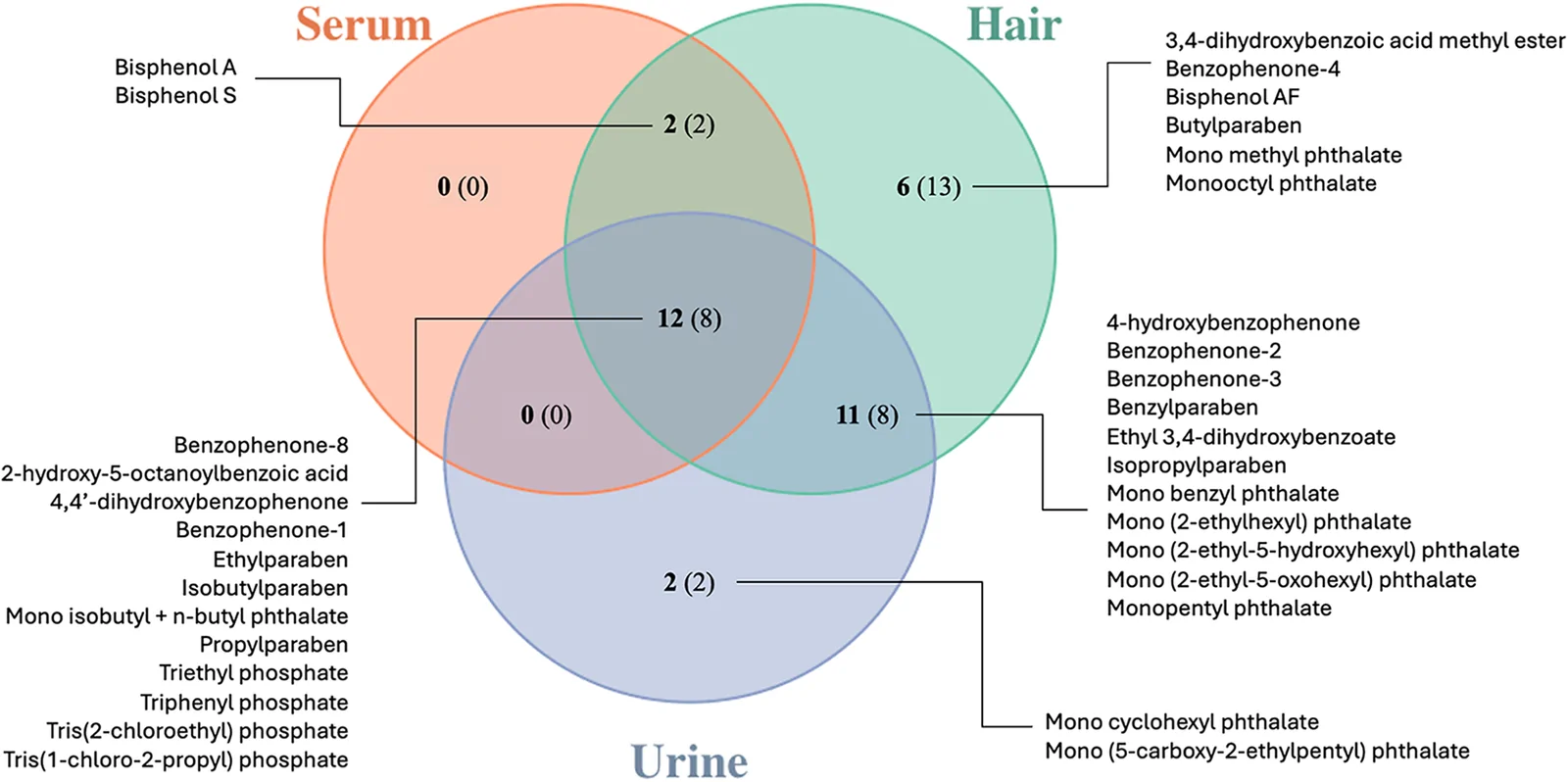
Venn diagram illustrating the number of chemicals detected in each matrix. **Bold numbers** indicate the total number of chemicals detected (either as their free or conjugated form). Numbers in brackets represent only the target chemicals, therefore excluding conjugates detected through suspect screening. Note that in some cases, numbers in brackets may exceed the bold totals because the shared chemicals across matrices are distributed differently for parent compounds versus conjugates. A detailed list of the parent compounds and phase II metabolites assigned to each Venn diagram region is provided in Table S7.

Detection frequencies differed markedly across matrices (Figure 3), being higher in hair for most chemical families. For instance, 4,4′-dihydroxybenzophenone and 2-hydroxy-5-octanoylbenzoic acid were detected in 95% and 65% of hair samples, compared to 5% in serum and 40% and 5% in urine, respectively. This pattern underscores hair’s ability to integrate longer-term exposure signals, reflecting cumulative uptake from systemic circulation and external deposition. In contrast, serum and urine showed lower and less consistent detection frequencies, reflecting short-term exposures. Phthalate metabolites represented the main exception, showing higher detection frequencies in urine than in hair, consistent with their rapid biotransformation and urinary excretion. This likely reflects that incorporation into hair requires systemic transport and deposition into the growing hair shaft, which occurs more slowly than urinary elimination.^72^ A few compounds, including triethyl phosphate, triphenyl phosphate, and mono butyl phthalate, displayed slightly higher detection frequencies in serum than in hair, likely influenced by matrix-specific LOQs rather than true absence. From our target list, 12 were detected in all three matrices, facilitating a comprehensive comparison of their behavior. All of them have been individually reported in serum, hair, and urine in previous studies.^45,73–76^ Among these chemicals, mono butyl phthalate exhibited high detection frequencies across all matrices (95% in hair, 85% in urine, and 100% in serum). These results align with previous studies reporting its ubiquitous presence in biological samples.^77–79^

**Figure 3:**
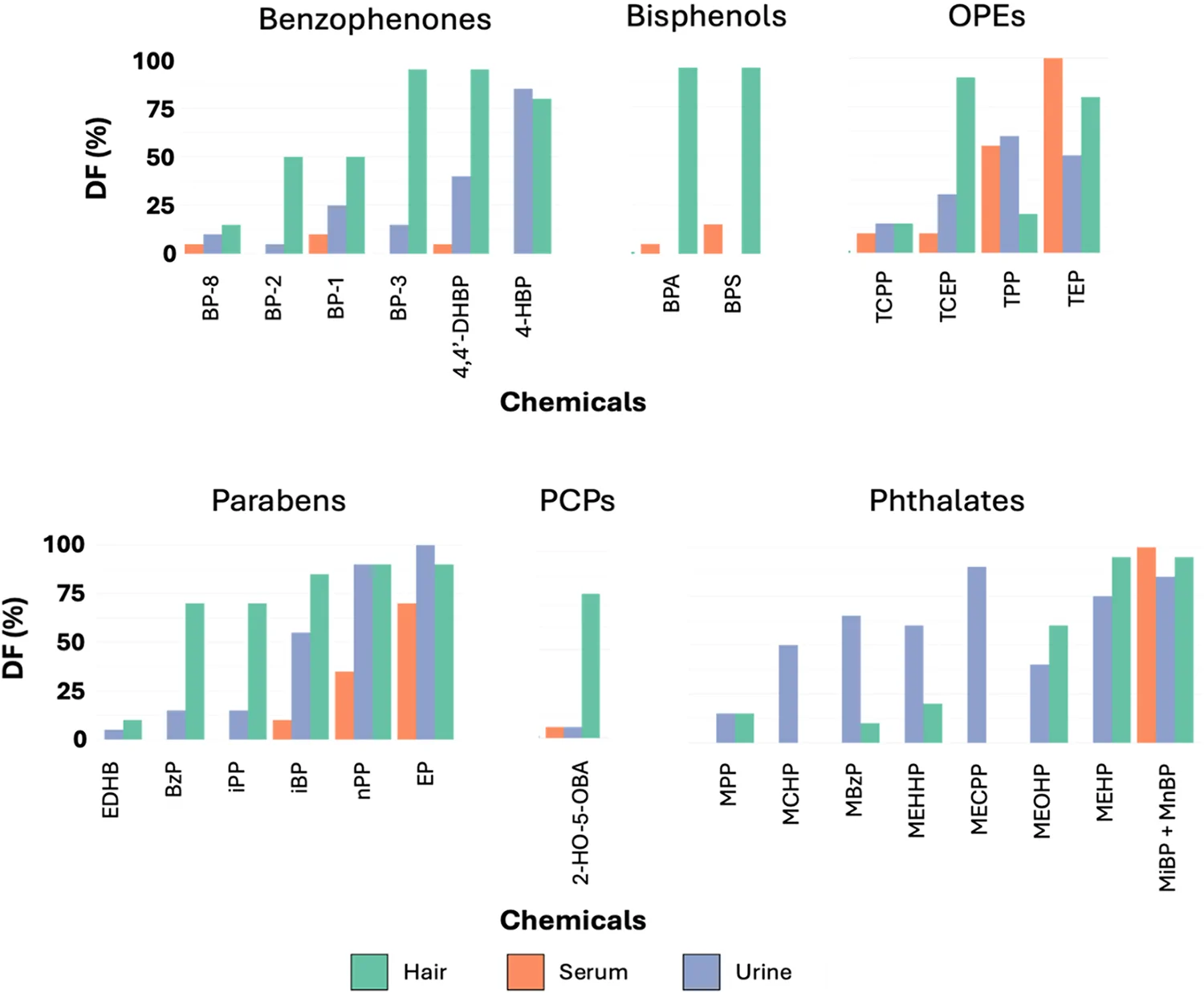
Detection frequencies (%) of the chemicals detected in at least two matrices. The bars indicate the detection frequency (DF, %) for each matrix. Chemicals detected in only one matrix are not shown. BP-1: benzophenone-1; BP-2: benzophenone-2; BP-3: benzophenone-3; BP-8: benzophenone-8; 4,4′-DHBP: 4,4′-dihydroxybenzophenone; 4-HBP: 4-hydroxybenzophenone; BPA: bisphenol A; BPS: bisphenol S; TCEP: tris (2-chloroethyl) phosphate; TCPP: tris (1-chloro-2-propyl) phosphate; TPP: triphenyl phosphate; TEP: triethyl phosphate; EDHB: ethyl 3,4-dihydroxybenzoate; BzP: benzyl paraben; iPP: isopropylparaben; iBP: isobutyl paraben; nPP: propyl paraben; EP: ethyl paraben; 2-HO-5-OBA: 2-hydroxy-5-octanoylbenzoic acid; MPP: mono pentyl phthalate; MCHP: mono cyclohexyl phthalate; MBzP: mono benzyl phthalate; MEHHP: mono 2-ethyl-5-hydroxyhexyl phthalate; MECPP: mono 5-carboxy-2-ethylpentyl phthalate; MEOHP: mono 2-ethyl-5-oxohexyl phthalate; MEHP: mono 2-ethylhexyl phthalate; MiBP + MnBP: mono isobutyl phthalate + mono *n*-butyl phthalate.

Concentration profiles differed substantially among biospecimens (Figure 4 and Table S9). Hair, urine, and serum represent distinct biological compartments with different incorporation mechanisms, elimination kinetics, temporal integration windows, and concentration units. Consequently, observed concentration differences may reflect both exposure characteristics and matrix-specific toxicokinetic processes. While hair integrates exposure over several months, serum and urine primarily reflect recent exposure events and ongoing elimination processes. Despite these differences, hair concentrations were typically one to several orders of magnitude higher than those measured in urine or serum on a relative scale. Within each matrix, median total concentrations ranged from 0.5 to 880.2 ng/g in hair, 0.2 to 81.9 μg/g creatinine in urine, and 0.2 to 25.9 ng/mL in serum. This interpretation is further supported by the greater distance of measured concentrations from detection limits in hair, where minimum detected values were on average ∼20-fold above the LOD, compared to ∼7-fold in urine and ∼4-fold in serum, indicating not only higher concentrations but also a reduced influence of censoring near detection limits. Hair showed greater interindividual variability, reflecting the accumulation of both low- and high-level exposures over time. In contrast, serum and urine exhibited narrower distributions, consistent with their role as short-term matrices capturing transient exposure events. Exceptions were benzophenone-8 in serum and benzophenone-1 and 4,4′-dihydroxybenzophenone in urine, which showed elevated variability (Figures S1–S3). Together, these patterns highlight hair’s unique ability to integrate exposure fluctuations and extend the temporal resolution of EDCs biomonitoring.

**Figure 4:**
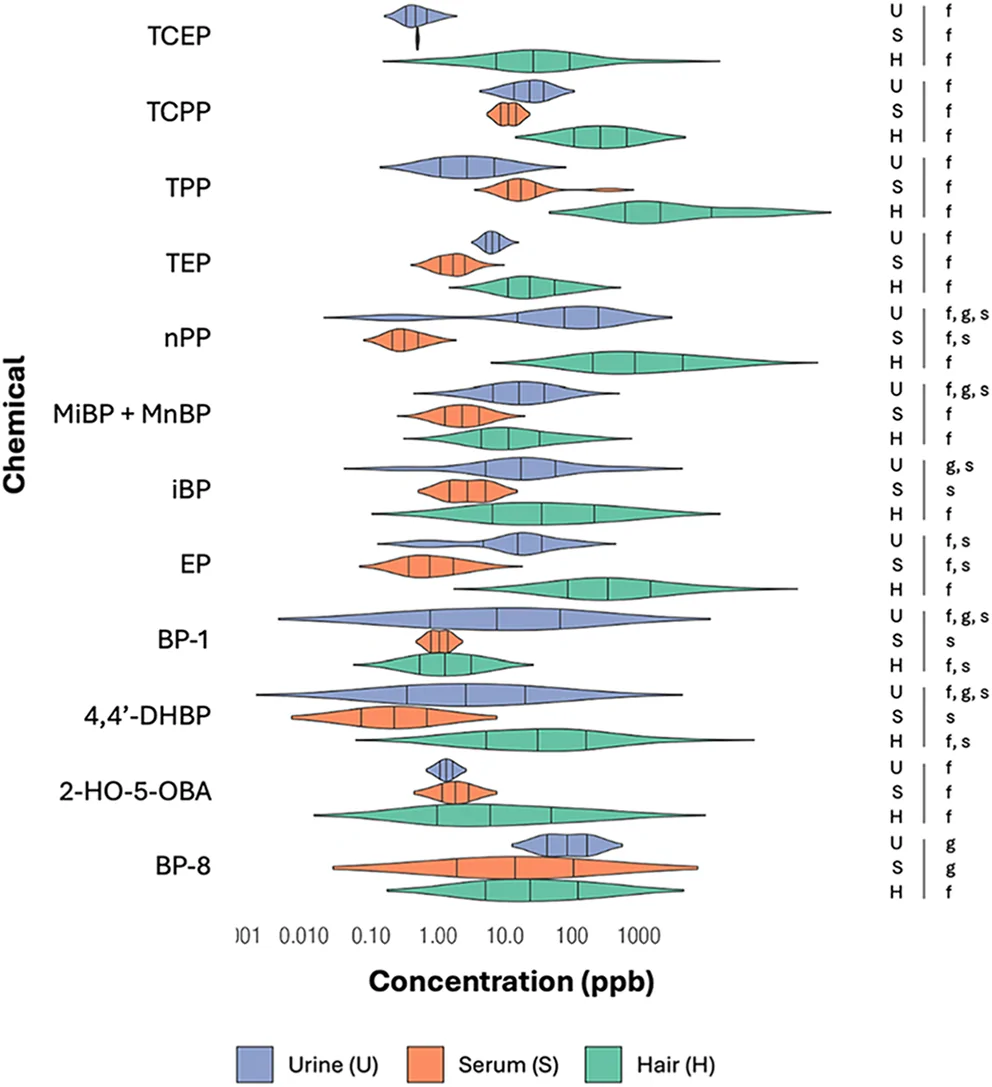
Total logarithmic concentration distribution of the 12 chemicals detected across all three matrices. The letters on the right indicate the contribution of biomarkers to the total concentration: f (free form), g (glucuronide form), and s (sulfate form). The matrices are indicated as follows: U (urine), S (serum), and H (hair). BP-1: benzophenone-1; BP-8: benzophenone-8; 4,4′-DHBP: 4,4′-dihydroxybenzophenone; TCEP: tris (2-chloroethyl) phosphate; TCPP: tris (1-chloro-2-propyl) phosphate; TPP: triphenyl phosphate; TEP: triethyl phosphate; iBP: isobutyl paraben; nPP: propyl paraben; EP: ethyl paraben; 2-HO-5-OBA: 2-hydroxy-5-octanoylbenzoic acid; MiBP + MnBP: mono isobutyl phthalate + mono *n*-butyl phthalate.

In addition, 11 chemicals, three benzophenones, three parabens, and five phthalate metabolites were detected in both hair and urine samples but not in serum. While benzophenones, parabens, and phthalate metabolites have been previously identified individually in both matrices,^36,80,81^ ethyl 3,4-dihydroxybenzoate, commonly used as a preservative in cosmetics, was detected in hair samples and, to the best of our knowledge, has rarely been reported in this matrix, although it is a known urinary biomarker of paraben exposure.^82^ Their absence in serum likely reflects short half-lives in blood.^83^ Chemicals such as benzophenones and phthalates are rapidly metabolized and excreted in urine but may also gradually accumulate in long-term storage matrices, such as hair. Serum, in contrast, may only capture transient or low levels of these chemicals, making detection more challenging.^84^

Only two bisphenols, BPA and BPS, were detected in hair and serum, with notable differences in detection patterns across matrices. BPA showed high detection frequency in hair (95%) with a broad concentration range (6.03–272 ng/g), aligning with previous findings by Gonkowski et al. (72%) and Martín et al. (83%).^29,30,38^ In serum, BPA was detected in only one sample (25.9 ng/mL), highlighting its minimal presence in this matrix. BPS exhibited a similar trend: 95% detection in hair with a wide concentration range (10.0–1190 ng/g) compared to just 15% in serum with low levels (0.201–0.387 ng/mL). These findings confirm that hair effectively captures long-term exposure to BPA and BPS, whereas serum reflects only short-term or recent exposure.^85^ Neither BPA nor BPS was detected in urine, despite being primarily excreted in this matrix as glucuronide and sulfate conjugates.^86^ This absence may partly reflect the limited sensitivity of the analytical method for these compounds in urine (LOQ = 0.5 ng/mL) and the applied blank correction procedures, as reported median urinary concentrations are approximately 1.2 ng/mL for BPA and 0.17 ng/mL for BPS,^87,88^ values that are close to or below our detection limits. While our extraction protocol was optimized for broad chemical coverage across matrices, this trade-off resulted in limited sensitivity for certain analytes in urine.

However, the near-ubiquitous detection of BPA and BPS in hair (19 of 20 samples) together with their complete absence from the paired urine samples is difficult to attribute solely to differences in analytical sensitivity. A potential contribution from exogenous uptake, where chemicals from personal care products or ambient air partition directly into the internal hair shaft, cannot be excluded, even after applying a stringent solvent-based decontamination procedure prior to extraction (see Section 4).

Nevertheless, the use of spot urine samples may partly explain the apparent discrepancy. Spot urine samples primarily reflect recent exposure occurring over the preceding hours to approximately 1 day, whereas hair integrates exposure over a period of several months. Consequently, the longer integration window provided by hair increases the likelihood of detecting compounds resulting from intermittent or variable exposure and may better reflect the average exposure levels over time.

Notably, all chemicals detected in serum and urine were also found in hair, with the sole exceptions being the phthalate metabolites mono cyclohexyl phthalate (MCHP) and mono 5-carboxy-2-ethylpentyl phthalate (MECPP), which were present only in urine. Six chemicals were detected exclusively in hair, namely butyl paraben, a paraben metabolite (3,4-dihydroxybenzoic acid methyl ester), benzophenone-4, two phthalates (mono methyl phthalate and mono octyl phthalate), and bisphenol AF. Given that hair reflects cumulative exposure, with each cm representing about one month of exposure, these detections likely reflect earlier exposure events.^89^ The capacity of hair to bioconcentrate lipophilic compounds facilitates their detection even when circulating levels fall below quantifiable limits in blood or urine. Two chemicals were detected solely in urine: MCHP and MECPP. Both have been previously reported in this matrix, with concentration ranges comparable to those observed in this study.^90^ MCHP, a metabolite of DCHP, is not commonly detected due to its low concentrations, but was observed in 50% of the samples, with concentrations ranging from 0.561 to 36.0 μg/g creatinine, aligning with earlier reports.^91,92^ MECPP, a key metabolite for assessing DEHP exposure, was detected at concentrations between 12.9 and 88.9 μg/g creatinine, somewhat lower than previously reported ranges of 4.8 to 1112 μg/g creatinine.^93,94^

To explore factors influencing matrix partitioning, we examined correlations between hair-to-serum concentration ratios and physicochemical properties for chemicals detected in both matrices (*n* = 14). Molecular size descriptors showed no significant correlation with hair-to-serum ratios (Figure S6), and all 95% confidence intervals were wide and crossed zero, indicating that none of the physicochemical descriptors examined reliably explained matrix partitioning within this limited set of compounds. Inter-compound correlation analysis (Figure S7) revealed moderate to strong associations within chemical families, reflecting shared exposure sources and metabolic pathways. Organophosphate esters showed the strongest inter-correlations (TCEP-TEP: ρ = 0.76; TCEP-TPP: ρ = 0.64), consistent with their co-occurrence in flame-retardant formulations and household products. Phthalate metabolites derived from the same parent compound also correlated strongly (MEOHP-MECPP: ρ = 0.71; MEOHP-MEHP: ρ = 0.68), as expected from shared metabolic pathways. We acknowledge that the limited sample size reduces the statistical power of these correlations, so results should be interpreted cautiously. Nonetheless, the observed patterns underscore the value of multimatrix biomonitoring for capturing both individual chemical exposures and broader exposure profiles linked to product use.

### 3.3 Suspect Analysis of Metabolites

The additional suspect screening of endocrine disruptor metabolites enabled the identification of 26 conjugates not included in the target analysis. Of these 26 conjugates, most were found in urine (*n* = 24), as expected, since it is the main excretion route for biotransformation products. In contrast, only 6 conjugates were detected in serum and five in hair. Among the urinary metabolites, 15 were glucuronides and nine sulfates, while serum and hair showed a predominance of sulfate conjugates (only one glucuronide in each) (Figure S4).

After normalization of the semi-quantified concentrations, conjugated metabolites were estimated to contribute only a minor fraction of the total chemical burden in hair, indicating that EDCs were predominantly present in their free forms. Given the semi-quantitative nature of the approach, the exact estimated contribution of phase II metabolites should be considered, as the QSIR model’s uncertainty (within a factor of four for ∼80% of compounds) can slightly shift estimated contributions. Critically, this uncertainty does not affect the comparative pattern across matrices: the difference in conjugated-metabolite contribution between hair, serum, and urine spans more than an order of magnitude and is consistent with the distinct biological and metabolic properties of each matrix. As these metabolites are generated through endogenous biotransformation, their presence is consistent with biological incorporation into the growing hair shaft and supports the interpretation that at least part of the detected chemical burden reflects follicular incorporation rather than exclusively external deposition. The predominance of unconjugated compounds in hair is also consistent with the biological mechanisms governing chemical incorporation into this matrix. Hair incorporation occurs primarily during follicular growth through transfer from systemic circulation, whereas the mature keratinized shaft is metabolically inactive and lacks the enzymatic capacity for further biotransformation.^95^ In addition, phase II metabolites are generally more polar and hydrophilic than their parent compounds, reducing their affinity for the hair matrix and limiting their incorporation and retention. As a result, hair preferentially accumulates unconjugated species, whereas urine is enriched in conjugated metabolites reflecting active metabolic clearance.

Serum displayed a similar trend (free fraction ≈ 94%), except for a few chemicals, including benzophenone-8, 4,4′-dihydroxybenzophenone, benzophenone-1, and isobutyl paraben, which were detected only as phase II conjugates. In urine, seven chemicals were identified solely as metabolites: benzophenone-8, benzophenone-2, benzophenone-3, benzyl paraben, isobutyl paraben, isopropylparaben, and mono benzyl phthalate. All were detected as glucuronides, except isobutyl paraben and mono benzyl phthalate, which were mainly sulfated (88% and 31%, respectively). Regarding the contribution of phase II metabolites, their presence was confined to specific chemical classes. In serum, conjugates were observed only for parabens and benzophenones, while urine also contained glucuronidated phthalate metabolites. No metabolites were found for flame retardants, consistent with their higher stability and limited metabolic transformation.^96^

These results highlight the critical role of HRMS-based workflows for revealing metabolized forms that conventional targeted analyses would overlook. Boxplots illustrating the concentration distributions across matrices, grouped by chemical families, as well as the contribution of each metabolite to the total concentration, are shown in Figure S5.

Previous studies provide a useful context for these findings. Liao et al. analyzed free and conjugated forms of BPA in urine and serum using LC-MS/MS, finding that in urine samples, 32% corresponded to the free form, 7% to the sulfated form, and 57% to the glucuronidated form.^97^ In contrast, in serum, these ratios were 20, 34, and 46%, respectively. The small remaining percentage in urine was attributed to metabolites from the chlorinated derivatives. Similarly, Arbuckle et al. examined free and conjugated forms of BPA and triclosan in urine samples from pregnant women.^98^ The contribution of free BPA to the total concentration was minimal (1%), while the glucuronidated form contributed 90%, and the sulfated form 8.7%. These ratios contrast with the findings in this study, where BPA was not detected in urine in either free or conjugated form, and in serum, it was detected entirely in free form. These contrasts emphasize how metabolic profiles can differ by the matrix, population, and exposure scenario. Feng et al. analyzed both phase I and phase II metabolites of phthalates in urine samples from fertile and infertile men, focusing primarily on the glucuronidated forms as the main phase II metabolites of phthalates.^99^ Although some ratios differed from those reported in this study (such as for mono 2-ethyl-5-hydroxyhexyl phthalate and mono 2-ethyl-5-oxohexyl phthalate), the majority of metabolites showed similar ratios. For instance, the study reported a 6% free form for mono benzyl phthalate, while this form did not contribute to the total concentration in this cohort. Likewise, the free form of mono 2-ethylhexyl phthalate was reported at 11%, compared to 28% in this study. Table S8 presents the contributions of the free and conjugate forms to the total concentration of each chemical across the different matrices. Overall, this analysis underscores the complementarity of multimatrix profiling: hair predominantly reflects long-term accumulation of unmetabolized compounds with high medium- and long-term reproducibility, serum captures transient free species at short-term exposure, and urine records active metabolic clearance also from the short-term exposome. Such integrated profiling is essential to comprehensively characterizing human internal exposure and understanding how metabolic transformation shapes the persistence and detectability of endocrine disruptors across biological compartments.

### 3.4 Illustrative Application: Endocrine Disruptor Profiles in a Frontal Fibrosing Alopecia Cohort

As an illustrative exploratory case, the validated workflow was applied to paired hair, serum, and urine samples from 20 postmenopausal women, including 10 diagnosed with frontal fibrosing alopecia (FFA) and 10 age-matched controls. This small data set was used solely to illustrate real-world variability in exposure profiles and matrix complementarity, not to draw epidemiological inferences. Hair showed the broadest chemical coverage (31 compounds) and the highest overall EDC concentrations, whereas serum provided limited chemical information (14 compounds), and urine mainly reflected recent exposures (25 compounds). In total, 21 chemicals showed higher median concentrations in FFA cases and 11 in controls, a pattern consistent across all EDC families. Table 1 summarizes the concentration data by group, showing that median concentrations of several parabens, bisphenols, and flame retardants were higher in the FFA subgroup, suggesting possible differences in cumulative exposure patterns. Differences in detection frequencies were also observed, with triphenyl phosphate detected in 90% of cases versus 30% of controls and mono­(2-ethyl-5-oxohexyl) phthalate in 70% versus 10%, respectively. These differences were most notable for bisphenol S and selected organophosphate esters, such as triphenyl phosphate and tris­(1-chloro-2-propyl) phosphate. Given the emerging evidence that EDCs may play a role in hormone-related or immune-mediated disorders, including FFA,^100,101^ these descriptive patterns may reflect differences in underlying exposure profiles between cases and controls. However, given the small sample size (*n* = 10 per group), these findings are purely descriptive and hypothesis-generating rather than inferential.

**Table 1:** Concentrations of Chemicals Detected in Serum, Hair, and Urine, Grouped by Case, Control, and Overall^a^

	Serum (ng/mL)						Hair (ng/g)						Urine (μg/g creatinine)					
	Overall (*n* = 20)		Case (*n* = 10)		Control (*n* = 10)		Overall (*n* = 20)		Case (*n* = 10)		Control (*n* = 10)		Overall (*n* = 20)		Case (*n* = 10)		Control (*n* = 10)	
Chemical	Median (IQR)	DF (%)	Median (IQR)	DF (%)	Median (IQR)	DF (%)	Median (IQR)	DF (%)	Median (IQR)	DF (%)	Median (IQR)	DF (%)	Median (IQR)	DF (%)	Median (IQR)	DF (%)	Median (IQR)	DF (%)
2-hydroxy-5-octanoylbenzoic acid	1.83 (NA)	5	ND		1.83 (NA)	10	3.59 (41.0)	65	2.80 (63.9)	70	5.92 (8.41)	60	1.33 (NA)	5	ND		1.33 (NA)	10
3,4-dihydroxybenzoic acid methyl ester	ND		ND		ND		20.1 (67.6)	85	29.6 (123)	100	10.6 (38.4)	70	ND		ND		ND	
4,4′-dihydroxybenzophenone	0.223 (NA)	5	ND		0.223 (NA)	10	37.3 (104)	95	97.2 (137)	100	37.0 (46.5)	90	2.54 (13.4)	40	7.68 (16.1)	60	0.468 (0.362)	20
Benzyl paraben	ND		ND		ND		3.00 (3.14)	70	3.67 (1.46)	70	1.05 (2.69)	70	48.2 (47.7)	15	5.54 (NA)	10	74.6 (26.4)	20
Isobutyl paraben	3.33 (1.83)	10	ND		3.33 (1.83)	20	51.6 (174)	85	18.2 (174)	90	53.1 (214)	80	21.9 (29.3)	55	25.0 (18.9)	70	7.57 (166)	40
Isopropylparaben	ND		ND		ND		3.96 (7.21)	70	7.62 (17.0)	80	3.26 (1.60)	60	14.8 (26.1)	15	66.7 (NA)	10	14.7 (0.164)	20
Butyl paraben	ND		ND		ND		68.9 (219)	95	81.2 (137)	100	68.9 (282)	90	ND		ND		ND	
4-hydroxybenzophenone	ND		ND		ND		2.70 (10.4)	80	2.37 (9.42)	90	3.04 (11.8)	70	73.7 (121)	85	118 (118)	100	71.4 (101)	70
Benzophenone-1	1.10 (0.309)	10	0.788 (NA)	10	1.41 (NA)	10	1.20 (2.47)	50	1.74 (3.43)	50	0.966 (1.04)	50	20.3 (24.8)	25	0.736 (12.5)	30	64.3 (44.0)	20
Benzophenone-2	ND		ND		ND		5.58 (12.2)	50	7.02 (5.09)	50	4.15 (13.6)	50	120 (NA)	5	ND		120 (NA)	10
Benzophenone-3	ND		ND		ND		56.3 (396)	95	36.2 (1440)	100	69.5 (241)	90	53.4 (53.0)	15	6.03 (NA)	10	82.7 (29.3)	20
Benzophenone-4	ND		ND		ND		51.2 (222)	60	68.2 (157)	70	17.7 (1080)	50	ND		ND		ND	
Benzophenone-8	14.4 (NA)	5	ND		14.4 (NA)	10	20.6 (98.6)	15	20.6 (98.6)	30	ND		106 (63.5)	10	ND		106 (63.5)	20
Bisphenol A	25.9 (NA)	5	ND		25.9 (NA)	10	67.7 (42.0)	95	59.7 (38.1)	100	70.3 (47.1)	90	ND		ND		ND	
Bisphenol AF	ND		ND		ND		15.1 (NA)	5	ND		15.1 (NA)	10	ND		ND		ND	
Bisphenol S	0.246 (0.093)	15	ND		0.246 (0.093)	30	119 (298)	95	220 (300)	100	67.8 (115)	90	ND		ND		ND	
Ethyl 3,4-dihydroxybenzoate	ND		ND		ND		22.6 (12.2)	10	22.6 (12.2)	20	ND		0.954 (NA)	5	ND		0.954 (NA)	10
Ethyl paraben	0.603 (1.19)	70	0.515 (0.603)	60	0.946 (1.18)	80	317 (1116)	75	258 (1470)	70	433 (965)	80	15.5 (17.2)	100	24.3 (20.9)	100	11.9 (14.2)	100
Mono benzyl phthalate	ND		ND		ND		25.2 (23.4)	10	25.2 (23.4)	20	ND		7.30 (6.11)	65	8.77 (5.02)	60	7.04 (6.01)	70
Mono cyclohexyl phthalate	ND		ND		ND		ND		ND		ND		7.28 (11.0)	50	5.99 (9.62)	60	11.9 (17.2)	40
Mono iso + *n*-butyl phthalate	2.33 (2.59)	100	2.29 (2.35)	100	2.58 (2.42)	100	11.0 (24.1)	95	11.3 (30.4)	100	11.0 (6.95)	90	19.4 (24.3)	85	10.3 (32.6)	90	19.6 (15.5)	80
Mono methyl phthalate	ND		ND		ND		166 (279)	55	47.3 (321)	50	221 (206)	60	ND		ND		ND	
Mono 2-ethyl-5-hydroxyhexyl phthalate	ND		ND		ND		4.54 (6.56)	20	7.50 (4.24)	30	1.58 (NA)	10	1.46 (5.59)	60	1.81 (5.33)	60	1.46 (3.95)	60
Mono 2-ethyl-5-oxohexyl phthalate	ND		ND		ND		2.81 (5.25)	60	1.99 (5.56)	60	3.45 (2.91)	60	1.15 (0.912)	40	1.32 (0.750)	70	0.413 (NA)	10
Mono 5-carboxy-2-ethylpentyl phthalate	ND		ND		ND		ND		ND		ND		36.3 (20.7)	90	37.9 (17.1)	100	33.0 (28.2)	80
Mono 2-ethylhexyl phthalate	ND		ND		ND		144 (269)	95	107 (327)	100	185 (143)	90	8.52 (8.81)	75	10.1 (11.7)	80	8.52 (8.63)	70
Mono octyl phthalate	ND		ND		ND		16.6 (10.0)	10	26.6 (NA)	10	6.66 (NA)	10	ND		ND		ND	
Mono pentyl phthalate	ND		ND		ND		0.526 (3.29)	15	6.93 (NA)	10	0.433 (0.093)	20	57.0 (42.8)	15	28.7 (28.3)	20	86.1 (NA)	10
Propyl paraben	0.289 (0.213)	35	0.229 (0.157)	30	0.333 (0.202)	40	880 (3940)	60	1320 (8270)	60	388 (1540)	60	70.0 (200)	90	119 (184)	100	60.4 (221)	80
Triphenyl phosphate	18.5 (11.7)	55	14.7 (13.5)	60	19.5 (3.99)	50	1660 (13700)	20	2350 (25200)	30	671 (NA)	10	3.33 (3.82)	60	3.91 (4.23)	90	1.41 (1.79)	30
Tris(2-chloroethyl) phosphate	0.5 (0.01)	10	ND		0.495 (0.011)	20	23.5 (61.0)	85	24.7 (94.1)	90	22.3 (44.7)	80	0.421 (0.241)	30	0.375 (0.105)	50	0.952 (NA)	10
Tris(1-chloro-2-propyl) phosphate	11.7 (3.02)	10	ND		11.7 (3.02)	20	271 (365)	15	544 (273)	20	87.8 (NA)	10	25.4 (14.3)	15	25.4 (NA)	10	25.9 (14.3)	20
Triethyl phosphate	1.90 (1.08)	100	2.16 (0.616)	100	1.10 (0.684)	100	23.7 (32.7)	80	21.8 (35.6)	80	23.7 (29.4)	80	6.30 (2.55)	50	6.30 (1.35)	40	6.73 (2.74)	60

a The table presents the median and interquartile range (IQR) of chemical concentrations in serum, hair, and urine samples for the case and control groups. The detection frequency (DF) is expressed as a percentage (%). ND indicates nondetected values, and NA denotes the absence of an interquartile range due to single observed values.

The higher variability observed in hair compared with serum or urine likely reflects its capacity to integrate both low- and high-intensity exposures over extended periods as well as interindividual factors such as hair growth rate, pigmentation, and use of personal care products. These matrix-dependent patterns illustrate the complementarity of hair, serum, and urine for integrating multiple temporal scales of exposure. While the observed differences align with hypotheses linking FFA to chronic exposure to cosmetic or UV-filter chemicals,^102,103^ the study was not designed to evaluate causality or perform statistical comparisons. Instead, this application demonstrates the practical value of the multimatrix LC-HRMS workflow for identifying interindividual differences and matrix-specific behaviors in real-world samples. It highlights how multimatrix exposomic profiling can generate hypothesis-driven insights into disease-related exposure patterns, even from limited sample sets, and guide the design of future targeted studies.

## 4 Implications and Limitations

This study demonstrates the analytical robustness and exposomic value of integrating hair into multimatrix biomonitoring frameworks for EDCs. Hair provided the broadest chemical coverage and the highest concentrations, capturing cumulative exposures that would otherwise escape detection in the serum or urine. By combining targeted and suspect screening of both free and conjugated species, the LC-HRMS workflow expanded the chemical space accessible to human biomonitoring and mitigated biases associated with enzyme-based or single-matrix approaches. The findings highlight that hair is particularly suited to assess long-term exposure to nonpersistent chemicals, complementing the more short-term information provided by serum and urine. This integrated strategy offers a more complete picture of exposure dynamics and strengthens the temporal and metabolic interpretation of the internal chemical burdens. However, hair is a complex and variable matrix. Interindividual differences in growth rate, pigmentation, melanin content, cosmetic treatments, and scalp or hair disorders (e.g., FFA) may influence chemical incorporation and therefore both temporal interpretation and quantitative comparison of hair concentrations. An additional limitation of hair biomonitoring is the difficulty of fully disentangling systemic incorporation from that of external contamination. While all samples underwent a solvent-based decontamination procedure prior to analysis, measured concentrations should be interpreted as reflecting a combination of biological incorporation and, for some compounds, residual external deposition. This consideration may be particularly relevant for chemicals commonly present in personal care products such as parabens and benzophenones.

Urinary concentrations were normalized by using creatinine, a widely used approach in human biomonitoring. However, creatinine excretion can vary with muscle mass and physiological status, particularly in postmenopausal women, and this variability could not be corrected for using specific gravity, as this alternative normalization measure was not available in this cohort. Our extraction protocol, optimized for broad chemical coverage, resulted in insufficient sensitivity for certain analytes in urine (e.g., BPA, BPS, triethyl phosphate), showing that some matrix-specific contrasts may be influenced by analytical factors, including differences in sensitivity, blank contamination, and matrix-specific limits of quantification, highlighting inherent trade-offs between multianalyte scope and compound-specific performance. Likewise, the limited number of participants precludes firm conclusions regarding interindividual or disease-related differences and restricts the statistical power to identify exposure-health associations. Despite these constraints, this study demonstrates that hair can serve as a valuable long-term exposomic matrix that complements conventional biospecimens, enabling the integration of temporal, metabolic, and physicochemical dimensions of exposure within the same analytical framework. Scaling this approach to larger and more diverse populations will help establish links between long-term chemical profiles and mechanistic and epidemiological outcomes. Ultimately, combining hair with conventional biospecimens can substantially enhance both the chemical and temporal resolution of human exposure assessment, thereby advancing the integration of exposomics into epidemiological research.

## Supplementary Material





## Notes

Chemicals and reagents; analytical and internal standards; QA/QC details; UPLC-QTOF parameters; method validation and suspect analysis data; contribution of free and conjugated forms across matrices; concentration data for detected endocrine disruptors; concentration distribution plots; metabolite occurrence and distribution; physicochemical correlations; and inter-compound correlation analyses; violin plot of the logarithmic concentration distribution for all chemicals, grouped by chemical families; violin plot of the logarithmic concentration distribution of chemicals detected only in one matrix; analytical standards used for method validation and quantification, and QTOF acquisition parameters (PDF)
Concentrations of detected endocrine disruptors across serum, hair, and urine (XLSX)
