# Supplementary material for: Exposomic Assessment of Endocrine-Disrupting Chemicals Using Hair: A Cross-Matrix Comparison with Serum and Urine by LC-HRMS and Metabolite Profiling
Source: Environ Sci Technol. 2026 Sep 11;60(37):26100–15. doi: 10.1021/acs.est.6c05029 (PMC13614180; doi:10.1021/acs.est.6c05029)
Supplement: Supplementary file 1 [file es6c05029_si_001.pdf]

Supplementary Material

Exposomic assessment of endocrine-disrupting chemicals using hair: a cross-matrix comparison with serum and urine by LC–HRMS and metabolite profiling

Ana González-Ruiz[1,2,5], Adrià Sunyer-Caldú[3], Rodrigo Braz Carneiro[4], Esteban Restrepo-Montes[5], Olivia Ballester-Miró[5], Alejandro Lobato-Berezo[6], Ramón Pujol[7], Montse Marquès[5], Rubén Gil-Solsona[5], Pablo Gago-Ferrero[5]*

[1]Laboratory of Toxicology and Environmental Health, School of Medicine, TecnATox, Universitat Rovira i Virgili (URV), Sant Llorenç 21, 43201 Reus, Spain

[2]Institut d'Investigació Sanitària Pere Virgili (IISPV), Doctor Mallafré Guasch 4, 43005 Tarragona, Spain

[3]Department of Environmental Science (ACES, Exposure & Effects), Science for Life Laboratory, Stockholm University, Stockholm, 106 91, Sweden

[4]São Carlos School of Engineering, University of São Paulo (EESC-USP), João Dagnone Ave 1100, São Carlos 13563-120, Brazil

[5]Department of Environmental Chemistry, Institute of Environmental Assessment and Water Research (IDAEA), Spanish Council of Scientific Research (CSIC), Jordi Girona 18-26, 08034 Barcelona, Spain

[6]Hospital Universitari Mútua Terrassa, Plaça Dr. Robert 5, 08221 Terrassa, Spain

[7]Institut Hospital del Mar, Passeig Marítim 25-29, 08003 Barcelona, Spain

<u>**Contents**</u>

Supplementary Sections

Section S1. Chemicals and reagents.

Section S2. QA/QC Protocols, Method Validation and Suspect Analysis.

Section S3. UPLC-QTOF parameters.

Tables

Table S1. Analytical standards used for method validation and quantification.

Table S2. Internal standards used for method validation and method performance evaluation.

Table S3. Gradient profile and flow rate changes for the LC method.

Table S4. QTOF acquisition parameters.

Table S5. Ionization mode, m/z, main fragments, and retention times of parent compounds and their corresponding phase II metabolites (glucuronides and sulfates) detected by suspect analysis.

Table S6. Hair extraction method validation parameters for all EDs analyzed.

Table S7. Distribution of parent compounds and phase II metabolites across Venn diagram regions.

Table S8. Contribution of free and conjugate forms to the total concentration of each chemical across the different matrices.

Table S9. Concentrations of detected endocrine disruptors across serum, hair, and urine. (In .xlsx separate file).

Figures

Figure S1. Violin plot of the logarithmic concentration distribution for all chemicals, grouped by chemical families.

Figure S2. Violin plot of the logarithmic concentration distribution of chemicals found in two matrices (hair-serum, hair-urine, or serum-urine).

Figure S3. Violin plot of the logarithmic concentration distribution of chemicals detected only in one matrix.

Figure S4. Venn diagram illustrating the number of metabolites detected in each matrix, along with the overlaps representing metabolites common to multiple matrices.

Figure S5. Boxplot showing the distribution of concentrations in each matrix grouped by families and the contribution of each metabolite to the total concentration.

Figure S6. Heatmap of Spearman correlations between the hair-to-serum ratio and physicochemical properties.

Figure S7. Heatmap of Spearman correlations among chemical compounds.

Supplementary Sections

Section S1. Chemicals and Reagents

Both analytical and internal standards (>95% purity) were purchased from LGC (Teddington, UK). Standard solutions were initially prepared at 1000 ng/mL in methanol and then diluted to 1 ng/mL. All solvents used were HPLC-grade, including acetonitrile (ACN) from Fisher (Waltham, USA), and methanol (MeOH), dichloromethane (DCM), and water from Merck (Darmstadt, Germany). Water and methanol for mobile phases were Optima® LC/MS grade by Fisher (Waltham, USA). Formic acid (98–100% purity), ammonium acetate (≥98.0% purity), and ammonium formate (≥99.0% purity) for the mobile phases were also purchased from Merck (Darmstadt, Germany). Milli-Q water was produced using a Milli-Q purification system Aurium PRO-VFT from Sartorius (Göttingen, Germany). Captiva 3 mL non-drip filter cartridges were bought from Agilent (Madrid, Spain), the creatinine kit was purchased from *Química Analítica Aplicada* (Tarragona, Spain), and 3 mm stainless-steel beads for bead beating were obtained from Werfen (Barcelona, Spain).

Section S2. QA/QC additional details.

All glassware was thoroughly rinsed with distilled water and acetone and then heated in a muffle furnace at 450 °C prior to use. Analytical and internal standards were kept in the dark in amber glass vials at −20°C to prevent degradation.

Section S3. UPLC-QTOF parameters

For positive ionization mode (+ESI), mobile phase consisted of an aqueous phase (A) of H_2O:MeOH (99:1) with 5 mM ammonium formate and 0.01% formic acid, and an organic phase (B) of MeOH with 5 mM ammonium formate and 0.01% formic acid. In negative ionization mode (-ESI), the mobile phase was an aqueous phase (A) of H_2O:MeOH (99:1) with 5 mM ammonium acetate, and an organic phase (B) of MeOH with 5 mM ammonium acetate. The total run time for the sequence was 20 minutes, with an injection volume of 10 µL.

Tables

Table S1. Native standards used for method validation and quantification.

Chemical	Ionization mode	Molecular formula	Chemical type	LogP[a]	CAS number
2-hydroxy-5-octanoylbenzoic acid	NEG	C15H20O4	Personal Care Product	5.2	78418-01-6
3,4-dihydroxybenzoic acid	NEG	C7H6O4	Parabens	1.1	99-50-3
3,4-dihydroxybenzoic acid methyl ester	NEG	C8H8O4	Parabens	1.5	2150-43-8
4,4'-dihydroxybenzophenone	NEG	C13H10O3	Benzophenones	2.7	611-99-4
Benzylparaben	NEG	C14H12O3	Parabens	3.6	94-18-8
Isobutylparaben	NEG	C11H14O3	Parabens	3.4	4247-02-3
Isopropylparaben	NEG	C10H12O3	Parabens	2.8	4191-73-5
4-hydroxybenzophenone	NEG	C13H10O2	Benzophenones	3.1	1137-42-4
Benzophenone-1	NEG	C13H10O3	Benzophenones	3.2	131-56-6
Benzophenone-2	NEG	C13H10O5	Benzophenones	2.4	131-55-5
Benzophenone-3	POS	C14H12O3	Benzophenones	3.6	131-57-7
Benzophenone-4	NEG	C14H12O6S	Benzophenones	2.2	4065-45-6
Benzophenone-8	NEG	C14H12O4	Benzophenones	3.3	131-53-3
Tetrabromo bisphenol A	NEG	C15H12Br4O2	Bisphenols	6.8	79-94-7
Bisphenol AP	NEG	C20H18O2	Bisphenols	4.4	1571-75-1
Bisphenol E	NEG	C14H14O2	Bisphenols	3.9	2081-08-5
Bisphenol G	NEG	C21H28O2	Bisphenols	6.3	127-54-8
Bisphenol M	NEG	C24H26O2	Bisphenols	6.1	13595-25-0
Bisphenol P	NEG	C24H26O2	Bisphenols	6.1	2167-51-3
Bisphenol Z	NEG	C18H20O2	Bisphenols	5.4	843-55-0
Bisphenol A	NEG	C15H16O2	Bisphenols	3.3	80-05-7
Bisphenol AF	NEG	C15H10F6O2	Bisphenols	4.5	1478-61-1
Bisphenol B	NEG	C16H18O2	Bisphenols	3.9	77-40-7
Bisphenol F	NEG	C13H12O2	Bisphenols	2.9	620-92-8
Bisphenol S	NEG	C12H10O4S	Bisphenols	1.9	80-09-1
Butylparaben	NEG	C11H14O3	Parabens	3.6	94-26-8
Ethyl 3,4-dihydroxybenzoate	NEG	C9H10O4	Parabens	1.8	3943-89-3
Ethylparaben	NEG	C9H10O3	Parabens	2.5	120-47-8
Mono benzyl phthalate	NEG	C15H12O4	Phthalates	3.3	2528-16-7
Mono cyclohexyl phthalate	NEG	C14H16O4	Phthalates	2.9	7517-36-4
Mono isobutyl + n-butyl phthalate	NEG	C12H14O4	Phthalates	2.8	131-70-4
Mono methyl phthalate	NEG	C9H8O4	Phthalates	1.1	4376-18-5
Mono(2-ethyl-5-hydroxyhexyl) phthalate	NEG	C16H22O5	Phthalates	2.5	40321-99-1
Mono(2-ethyl-5-oxohexyl) phthalate	NEG	C16H20O5	Phthalates	2.0	40321-98-0
Mono(5-carboxy-2-ethylpentyl) phthalate	NEG	C16H20O6	Phthalates	2.7	40809-41-4
Mono-2-ethylhexyl phthalate	NEG	C16H22O4	Phthalates	4.0	4376-20-9
Monooctyl phthalate	NEG	C16H22O4	Phthalates	5.3	5393-19-1
Monopentyl phthalate	NEG	C13H16O4	Phthalates	3.7	24539-56-8
Propylparaben	NEG	C10H12O3	Parabens	3.0	94-13-3
Triphenyl phosphate	POS	C18H15O4P	OPEs	4.6	115-86-6
Tris(2-chloroethyl) phosphate	POS	C6H12Cl3O4P	OPEs	1.3	115-96-8
Tris(1-chloro-2-propyl) phosphate	POS	C9H18Cl3O4P	OPEs	2.6	13674-84-5
Tri-o-tolyl phosphate	POS	C21H21O4P	OPEs	6.1	78-30-8
Triethyl phosphate	POS	C6H15O4P	OPEs	0.8	78-40-0

[a]LogP computed by XLogP3 3.0, PubChem.

Table S2. Internal standards used for method validation and method performance evaluation.

Chemical	Ionization mode	Molecular formula	Chemical type	LogP[a]	CAS number
2,6 di-(tertbutyl-d9)-4-Methyl-(phenol-3,5,O-d3)	NEG	C15D21H3O	IS	NA	64502-99-4
4-Methyl Benzylidene camphor-d4	POS	C18H18D4O	IS	3.8	1219806-41-3
Acetaminophen-d4	POS	C8D4H5NO2	IS	0.5	64315-36-2
Atenolol-d7	POS	C14D7H15N2O3	IS	0.2	1202864-50-3
Benzophenone-3-d5	POS	C14D5H7O3	IS	NA	1219798-54-5
Benzotriazole-d4	POS/NEG	C6HD4N3	IS	1.4	1185072-03-0
Benzyl paraben-d4	NEG	C14H8D4O3	IS	3.6	1219805-81-8
Caffeine-d3	POS	C8D3H7N4O2	IS	-0.1	26351-03-1
Carbamazepine-d10	POS	C15D10H2N2O	IS	2.5	132183-78-9
Citalopram-d4	POS	C20H18D4BrFN2O	IS	3.2	1219803-58-3
Diclofenac-d4 (phenyl-d4)	POS	C14D4H7Cl2NO2	IS	4.4	153466-65-0
Gemfibrozil-d6	NEG	C15D6H16O3	IS	3.8	1184986-45-5
Methyl paraben-d4	NEG	C8D4H4O3	IS	2.0	362049-51-2
Metronidazole-d4	POS	C6D4H5N3O3	IS	0	1261392-47-5
Nicotine-d3	POS	C10D3H11N2	IS	1.2	69980-24-1
Nonyl-phenol-d4	NEG	C15D4H20O	IS	5.9	1173019-62-9
Octylphenol-d17	NEG	C14D17H5O	IS	5.3	1219794-55-4
Stearic acid-13C18	NEG	13C18H36O2	IS	7.4	287100-83-8
Thiamethoxam-d3	POS	C8H7D3ClN5O3S	IS	1.5	1294048-82-0
Triphenyl phosphate-d15	POS	C18D15O4P	IS	4.6	1173020-30-8
Venlafaxine-d6	POS	C17D6H22NO2Cl	IS	2.9	1062606-12-5

[a]LogP computed by XLogP3 3.0, PubChem.

Table S3. Gradient profile and flow rate changes for the LC method.

Line	Time (min)	Flow rate (mL/min)	% A	% B
1	0.00	0.200	96.0	4.0
2	1.00	0.200	81.7	18.3
3	2.50	0.223	50.0	50.0
4	14.00	0.400	0.1	99.9
5	16.00	0.480	0.1	99.9
6	16.10	0.480	96.0	4.0
7	19.10	0.200	96.0	4.0
8	20.00	0.200	96.0	4.0

Table S4. QTOF acquisition parameters. *

Parameter	Value
**Capillary Voltage**	2500 V
**End Plate Offset**	500 V
**Nebulizer Pressure**	3 bar
**Drying Gas Flow Rate**	8 L/min
**Drying Gas Temperature**	200 °C
**Probe Gas Flow Rate**	4 L/min
**Probe Gas Temperature**	200 °C
**HRMS Mode**	Broadband Collision-Induced Dissociation (bbCID)
**Scan Rate**	3 scans per second
**MS Method Functions**	Low Energy and Ramp Energy
**Low Energy (LE) Collision Energy (+ESI)**	6 eV
**Low Energy (LE) Collision Energy (-ESI)**	8 eV
**Ramp Collision Energy (+ESI and -ESI)**	24–36 eV
**Scan Range (m/z)**	70–1000 Da
**Internal Calibration**	Sodium formate in first 30 seconds of each sample

* Source parameters correspond to standard acquisition settings recommended by Bruker for TargetScreener workflow and Intensity Solo column configuration and were applied in both ionization modes.

Table S5. Ionization mode, m/z, main fragments, and retention times of parent compounds and their corresponding phase II metabolites (glucuronides and sulfates) detected by suspect analysis. All suspect metabolites were assigned to Schymanski level 2b (probable structure by diagnostic evidence).

Metabolite	Ionization mode	m/z	Fragment 1	Fragment 2	Retention time
**2-hydroxy-5-octanoylbenzoic acid**	NEG	263.1284			9.2
**3,4-dihydroxybenzoic acid**	NEG	153.0188	109.0289		6.0
**3,4-dihydroxybenzoic acid methyl ester**	NEG	167.0344			5.3
**4-hydroxybenzophenone**	NEG	197.0603	121.0289		7.6
4-hydroxybenzophenone - glu	NEG	373.0923	197.0603		4.7
4-hydroxybenzophenone - SO_3	NEG	277.0177	197.0603		5.7
**4,4'-dihydroxybenzophenone**	NEG	213.0557	135.0087	93.0345	8.4
4,4'-dihydroxybenzophenone - glu	POS	391.1029	215.0714	121.0289	3.5
4,4'-dihydroxybenzophenone - SO_3	NEG	293.0126	213.0552	93.0345	4.9
**Benzophenone-1**	NEG	213.0557	135.0081	93.0345	5.9
Benzophenone-1 - glu	POS	391.1029	215.0714	137.0238	5.6
Benzophenone-1 - SO_3	NEG	293.0126	214.0636	93.0345	5.9
**Benzophenone-2**	NEG	245.0456	135.0081	91.0171	6.2
Benzophenone-2 - glu	POS	423.0927	247.0612	137.0238	4.0
**Benzophenone-3**	NEG	227.0714	136.0165		9.1
Benzophenone-3 - glu	NEG	403.1029	227.0714	136.0165	5.1
Benzophenone-3 - SO_3	NEG	307.0283	227.0714	136.0165	5.4
**Benzophenone-4**	NEG	307.0282	227.0708	211.0395	5.6
**Benzophenone-8**	NEG	243.0663			9.3
Benzophenone-8 - glu	POS	419.0978	245.0819		4.7
**Benzyl paraben**	NEG	227.0714	136.0160		9.2
Benzyl paraben - glu	POS	405.1185	229.0870		5.8
**Bisphenol A**	NEG	227.1078	211.0750	133.0659	8.1
**Bisphenol AF**	NEG	335.0512	265.0482	177.0343	9.3
**Bisphenol AP**	NEG	289.1229	273.0912	211.0748	9.4
**Bisphenol B**	NEG	241.1229	211.0759		8.9
**Bisphenol E**	NEG	213.0921			7.5
**Bisphenol F**	NEG	199.0759	93.0340		9.3
**Bisphenol G**	NEG	311.2012			11.5
**Bisphenol M**	NEG	345.1856			11.5
**Bisphenol P**	NEG	345.1856	330.1633	315.1386	11.7
**Bisphenol S**	NEG	249.0227	155.9881	108.0211	5.3
**Bisphenol Z**	NEG	267.1386	223.0751	145.0659	9.9
**Butyl paraben**	NEG	193.0865	137.0238	121.0306	9.4
**Ethyl 3,4-dihydroxybenzoate**	NEG	181.0501			6.0
**Ethyl paraben**	NEG	165.0552	137.0238		7.1
Ethyl paraben - SO_3	NEG	245.0126	165.0552	137.0238	5.0
**Isobutyl paraben**	NEG	193.0865	137.0238	121.0306	9.0
Isobutyl paraben - glu	NEG	369.1185	193.0865	137.0238	5.1
Isobutyl paraben - SO_3	NEG	273.0439	193.0865	137.0238	5.3
**Isopropyl paraben**	NEG	179.0708	93.0340		7.8
Isopropyl paraben - glu	POS	357.1185	181.0865		4.5
Isopropyl paraben - SO_3	NEG	259.0283	179.0708	93.0340	5.6
**Mono 2-ethyl-5-hydroxyhexyl phthalate**	NEG	293.1389	121.0289	77.0403	6.2
Mono 2-ethyl-5-hydroxyhexyl phthalate - glu	NEG	469.1710	293.1389	121.0289	5.2
**Mono 2-ethyl-5-oxohexyl phthalate**	NEG	291.1233	121.0289	143.1072	5.8
**Mono 2-ethylhexyl phthalate**	NEG	277.1440	121.0289	134.0367	9.3
Mono 2-ethylhexyl phthalate - glu	NEG	453.1760	277.1440	121.0289	5.7
**Mono 5-carboxy-2-ethylpentyl phthalate**	NEG	307.1182	121.0289		4.9
Mono 5-carboxy-2-ethylpentyl phthalate - SO_3	NEG	387.0756	307.1182	121.0289	4.3

Metabolite	Ionization mode	m/z	Fragment 1	Fragment 2	Retention time
**Mono benzyl phthalate**	NEG	255.0657	183.0810	121.0289	5.9
Mono benzyl phthalate - glu	NEG	431.0978	255.0657	183.0810	4.4
Mono benzyl phthalate - SO_3	NEG	335.0232	255.0657	183.0810	4.7
**Mono cyclohexyl phthalate**	NEG	247.0971	121.0289	95.0501	6.5
**Mono isobutyl + n-butyl phthalate**	NEG	221.0814	121.0289	71.0497	5.7
Mono isobutyl + n-butyl phthalate - glu	NEG	397.1134	221.0814	121.0289	3.9
Mono isobutyl + n-butyl phthalate - SO_3	NEG	301.0388	221.0814	121.0289	4.3
**Mono methyl phthalate**	NEG	179.0344	121.0289	107.0497	4.1
**Mono octyl phthalate**	NEG	277.1440	121.0295		9.6
**Mono pentyl phthalate**	NEG	235.0971	121.0295		6.7
Mono pentyl phthalate - glu	POS	413.1447	237.1127	149.0234	5.1
**Propyl paraben**	NEG	179.0708	136.0160	92.0262	8.3
Propyl paraben - glu	NEG	355.1029	179.0708	136.0160	4.0
Propyl paraben - SO_3	NEG	259.0283	179.0708	136.0160	4.3
**Tetrabromo bisphenol A**	NEG	538.7491	415.8087	78.9190	11.9
**Tri-o-tolyl phosphate**	POS	369.1257	166.0773	91.0544	12.9
**Triethyl phosphate**	POS	183.0787	98.9841	80.9737	6.2
**Triphenyl phosphate**	POS	327.0787	77.0387	152.0619	11.0
**Tris(1-chloro-2-propyl) phosphate**	POS	327.0089	233.0355	153.0687	9.3
**Tris(2-chloroethyl) phosphate**	POS	284.9619	98.9843	80.9744	6.9

Table S6. Hair extraction method validation parameters for all EDCs analyzed.

Chemical	Ionization mode	LOQ (ng/g)	Recoveries at 50 ng/g (%) ***	CV (%)	Linearity (R^2)	Matrix effect (%)	Procedural blanks (ng/g)	Concentration range (ng/g)
2-hydroxy-5-octanoylbenzoic acid	NEG	0.36	111.6	10.9	0.9932	74.0	0.1	0.36 - 250
3,4-dihydroxybenzoic acid	NEG	2	95.1	10.5	0.9982	71.1	0.9	2 - 250
3,4-dihydroxybenzoic acid methyl ester	NEG	0.4	71.3	8.1	0.9941	62.7	0.1	0.4 - 250
4,4'-dihydroxybenzophenone	NEG	0.2	109.9	4.2	0.9961	57.3	0.04	0.2 - 250
Benzylparaben	NEG	0.1	111.9	1.6	0.9973	46.7	0	0.1 - 250
Isobutylparaben	NEG	0.4	114.5	13.0	0.9815	10.0	0.02	0.4 - 250
Isopropylparaben	NEG	0.1	122.8	12.2	0.9944	7.7	0.01	0.1 - 250
4-hydroxybenzophenone	NEG	0.2	106.5	8.1	0.9962	58.1	0.04	0.2 - 250
Benzophenone-1	NEG	0.2	100.7	4.3	0.9984	28.5	0.04	0.2 - 250
Benzophenone-2	NEG	0.1	85.1	2.8	0.9991	22.9	0.04	0.1 - 250
Benzophenone-3	POS	4.3	83.0	5.8	0.9586	15.8	0.02	4.3 - 250
Benzophenone-4	NEG	0.5	352.4	25.4	0.9982	69.2	0.2	0.5 - 250
Benzophenone-8	NEG	2	87.0	9.4	0.9924	15.6	0.04	2 - 250
Bisphenol AP	NEG	1.9	81.8	4.6	0.9995	27.9	0	1.9 - 250
Bisphenol M	NEG	0.2	166.6	75.6	0.9917	17.0	0.2	0.2 - 250
Bisphenol P	NEG	1	62.3	6.4	0.9960	3.5	2.0	1 - 250
Bisphenol Z	NEG	10	102.5	11.9	0.9998	25.2	0	10 - 250
Bisphenol A	NEG	1.8	74.5	0.5	0.9973	61.1	0.7	1.8 - 250
Bisphenol AF	NEG	0.2	75.4	2.0	0.9964	51.7	0.3	0.2 - 250
Bisphenol B	NEG	2	106.1	1.0	0.9987	43.7	0	2 - 250
Bisphenol F	NEG	20	110.5	0.1	0.9999	19.9	0	20 - 250
Bisphenol S	NEG	2.4	65.6	6.9	0.9945*	18.9	0.2	2.4 - 250
Butylparaben	NEG	0.4	129.8	7.4	0.9866	15.4	0.03	0.4 - 250
Ethyl 3,4-dihydroxybenzoate	NEG	0.2	74.7	0.4	0.9973	67.4	0.2	0.2 - 250
Ethylparaben	NEG	5	117.5	10.9	0.9738	9.9	0.2	5 - 250
Mono benzyl phthalate	NEG	1	62.7	1.0	0.9996	68.6	0.02	1 - 250
Mono cyclohexyl phthalate	NEG	0.1	79.0	36.2	0.9989	82.3	0.02	0.1 - 250
Mono isobutyl + n-butyl phthalate	NEG	0.2	63.6	6.6	0.9987	83.1	0.2	0.2 - 250
Mono methyl phthalate	NEG	2.2	93.3	10.0	0.9999	87.5	1.0	2.2 - 250
Mono(2-ethyl-5-hydroxyhexyl) phthalate	NEG	0.08	94.1	7.1	0.9992	102.6	0.05	0.08 - 250
Mono(2-ethyl-5-oxohexyl) phthalate	NEG	0.05	84.1	4.3	0.9995	118.6	0.03	0.05 - 250
Mono(5-carboxy-2-ethylpentyl) phthalate	NEG	0.5	313.4	5.5	0.9999	31.8	0.1	0.5 - 250
Mono-2-ethylhexyl phthalate	NEG	3.8	88.7	1.9	0.9999	68.9	1.3	3.8 - 250
Monooctyl phthalate	NEG	0.4	72.1	11.1	0.9964	21.4	0.04	0.4 - 250
Monopentyl phthalate	NEG	0.1	73.0	8.5	0.9982	88.2	0.1	0.1 - 250
Propylparaben	NEG	7.8	98.2	10.1	0.9707	11.8**	0.5	7.8 - 250
Triphenyl phosphate	POS	67.2	29.8	0.9	0.9990*	5.7	28.4	67.2 - 250
Tris(2-chloroethyl) phosphate	POS	0.4	95.0	0.4	0.9992	10.3	0.3	0.4 - 250
Tris(1-chloro-2-propyl) phosphate	POS	5.3	79.8	1.3	0.9982*	18.7	0	5.3 - 250
Tri-o-tolyl phosphate	POS	1	43.9	2.2	0.9935	21.4	0.03	1 - 250
Triethyl phosphate	POS	0.8	76.4	1.7	0.9999	2.8	0.1	0.8 - 250

* Linearity of solvent calibration curves

** Matrix effect calculated as A CC 50 ng/mL hair / A CC 50 ng/mL solvent · 100 (the rest has been made comparing the slopes)

*** Recovery experiments and LOQ determinations were performed using different pooled hair samples. Consequently, differences in background concentrations may result in recovery experiments being conducted at fortification levels below the LOQ estimated from the matrix-matched calibration curves (e.g., triphenyl phosphate).

Table S7. Distribution of parent compounds and phase II metabolites across Venn diagram regions. Compounds were assigned to mutually exclusive regions according to the biological matrix or combination of matrices in which they were detected.

Venn region parent (brackets)	Venn region parent + metabolite (bold)	Chemical	Detection type
Hair only	Hair only	3,4-dihydroxybenzoic acid methyl ester	Parent compounds
Hair only	Hair + Urine	Benzophenone-2	Parent compounds
Hair only	Hair + Urine	Benzophenone-3	Parent compounds
-	Hair + Urine	Benzophenone-3 - SO_3	Phase II metabolites
Hair only	Hair only	Benzophenone-4	Parent compounds
Hair only	All three matrices	Benzophenone-8	Parent compounds
Hair only	Hair + Urine	Benzylparaben	Parent compounds
Hair only	Hair only	Bisphenol AF	Parent compounds
Hair only	Hair only	Butylparaben	Parent compounds
Hair only	All three matrices	Isobutylparaben	Parent compounds
Hair only	Hair + Urine	Isopropylparaben	Parent compounds
-	Hair + Urine	Isopropyl paraben - SO_3	Phase II metabolites
Hair only	Hair + Urine	Mono benzyl phthalate	Parent compounds
Hair only	Hair only	Mono methyl phthalate	Parent compounds
Hair only	Hair only	Monooctyl phthalate	Parent compounds
Urine only	Urine only	Mono cyclohexyl phthalate	Parent compounds
Urine only	Urine only	Mono(5-carboxy-2-ethylpentyl) phthalate	Parent compounds
-	Urine only	Mono(5-carboxy-2-ethylpentyl) phthalate - SO_3	Phase II metabolites
-	Hair + Urine	4-hydroxybenzophenone - glu	Phase II metabolites
-	Hair + Urine	4-hydroxybenzophenone - SO_3	Phase II metabolites
-	All three matrices	4,4'-dihydroxybenzophenone - glu	Phase II metabolites
-	All three matrices	Benzophenone-1 - glu	Phase II metabolites
-	Hair + Urine	Benzophenone-2 - glu	Phase II metabolites
-	Hair + Urine	Benzyl paraben - glu	Phase II metabolites
-	All three matrices	Isobutyl paraben - glu	Phase II metabolites
-	Hair + Urine	Isopropyl paraben - glu	Phase II metabolites
-	Hair + Urine	Mono 2-ethyl-5-hydroxyhexyl phthalate - glu	Phase II metabolites
-	Hair + Urine	Mono 2-ethylhexyl phthalate - glu	Phase II metabolites
-	Hair + Urine	Mono benzyl phthalate - glu	Phase II metabolites
-	Hair + Urine	Mono benzyl phthalate - SO_3	Phase II metabolites
-	All three matrices	Mono isobutyl + n-butyl phthalate - glu	Phase II metabolites
-	All three matrices	Mono isobutyl + n-butyl phthalate - SO_3	Phase II metabolites
-	Hair + Urine	Mono pentyl phthalate - glu	Phase II metabolites
-	All three matrices	Propyl paraben - glu	Phase II metabolites
Serum + Hair	Serum + Hair	Bisphenol A	Parent compounds
Serum + Hair	Serum + Hair	Bisphenol S	Parent compounds
Hair + Urine	All three matrices	4,4'-dihydroxybenzophenone	Parent compounds
Hair + Urine	Hair + Urine	4-hydroxybenzophenone	Parent compounds
Hair + Urine	All three matrices	Benzophenone-1	Parent compounds
Hair + Urine	Hair + Urine	Ethyl 3, 4-dihydroxybenzoate	Parent compounds
Hair + Urine	Hair + Urine	Mono(2-ethyl-5-hydroxyhexyl) phthalate	Parent compounds
Hair + Urine	Hair + Urine	Mono(2-ethyl-5-oxohexyl) phthalate	Parent compounds
Hair + Urine	Hair + Urine	Mono-2-ethylhexyl phthalate	Parent compounds
Hair + Urine	Hair + Urine	Monopentyl phthalate	Parent compounds
-	Hair + Urine	Benzophenone-3 - glu	Phase II metabolites
All three matrices	All three matrices	2-hydroxy-5-octanoylbenzoic acid	Parent compounds
All three matrices	All three matrices	Ethylparaben	Parent compounds
All three matrices	All three matrices	Mono isobutyl + n-butyl phthalate	Parent compounds
All three matrices	All three matrices	Propylparaben	Parent compounds
All three matrices	All three matrices	Triphenyl phosphate	Parent compounds
All three matrices	All three matrices	Tris(2-chloroethyl) phosphate	Parent compounds
All three matrices	All three matrices	Tris(1-chloro-2-propyl) phosphate	Parent compounds
All three matrices	All three matrices	Triethyl phosphate	Parent compounds

-	All three matrices	4,4'-dihydroxybenzophenone - SO_3	Phase II metabolites
-	All three matrices	Benzophenone-1 - SO_3	Phase II metabolites
-	All three matrices	Benzophenone-8 - glu	Phase II metabolites
-	All three matrices	Ethyl paraben - SO_3	Phase II metabolites
-	All three matrices	Isobutyl paraben - SO_3	Phase II metabolites
-	All three matrices	Propyl paraben - SO_3	Phase II metabolites

Table S8. Contribution of free and conjugate forms to the total concentration of each chemical across the different matrices. Values are expressed as percentages. Only chemicals with detected phase II metabolites are reported, as the contribution of the free form for the remaining chemicals is 100%.

Chemical	Hair			Serum			Urine		
	Free	Glucuronide	Sulfate	Free	Glucuronide	Sulfate	Free	Glucuronide	Sulfate
**4-hydroxybenzophenone**	100	-	-	-	-	-	0.2	1	98.8
**4,4'-dihydroxybenzophenone**	98	-	2	-	-	100	1	93.5	5.5
**Benzophenone-1**	97.2	-	2.8	-	-	100	0.3	98.3	1.4
**Benzophenone-2**	100	-	-	-	-	-	-	100	-
**Benzophenone-3**	99.5	0.1	0.4	-	-	-	-	100	-
**Benzophenone-8**	100	-	-	-	100	-	-	100	-
**Benzyl paraben**	100	-	-	-	-	-	-	100	-
**Ethyl paraben**	100	-	-	2.5	-	97.5	3.1	-	96.9
**Isobutyl paraben**	100	-	-	-	-	100	-	12.1	87.9
**Isopropyl paraben**	99.7	-	0.3	-	-	-	-	100	-
**Mono 2-ethyl-5-hydroxyhexyl phthalate**	100	-	-	-	-	-	96.3	3.7	-
**Mono 2-ethylhexyl phthalate**	100	-	-	-	-	-	28.4	71.6	-
**Mono 5-carboxy-2-ethylpentyl phthalate**	-	-	-	-	-	-	91.2	-	8.8
**Mono benzyl phthalate**	100	-	-	-	-	-	-	69.2	30.8
**Mono isobutyl + n-butyl phthalate**	100	-	-	100	-	-	25.8	11.8	62.4
**Mono pentyl phthalate**	100	-	-	-	-	-	0.3	99.7	-
**Propyl paraben**	100	-	-	34.5	-	65.5	0.1	2.2	97.7

Figures

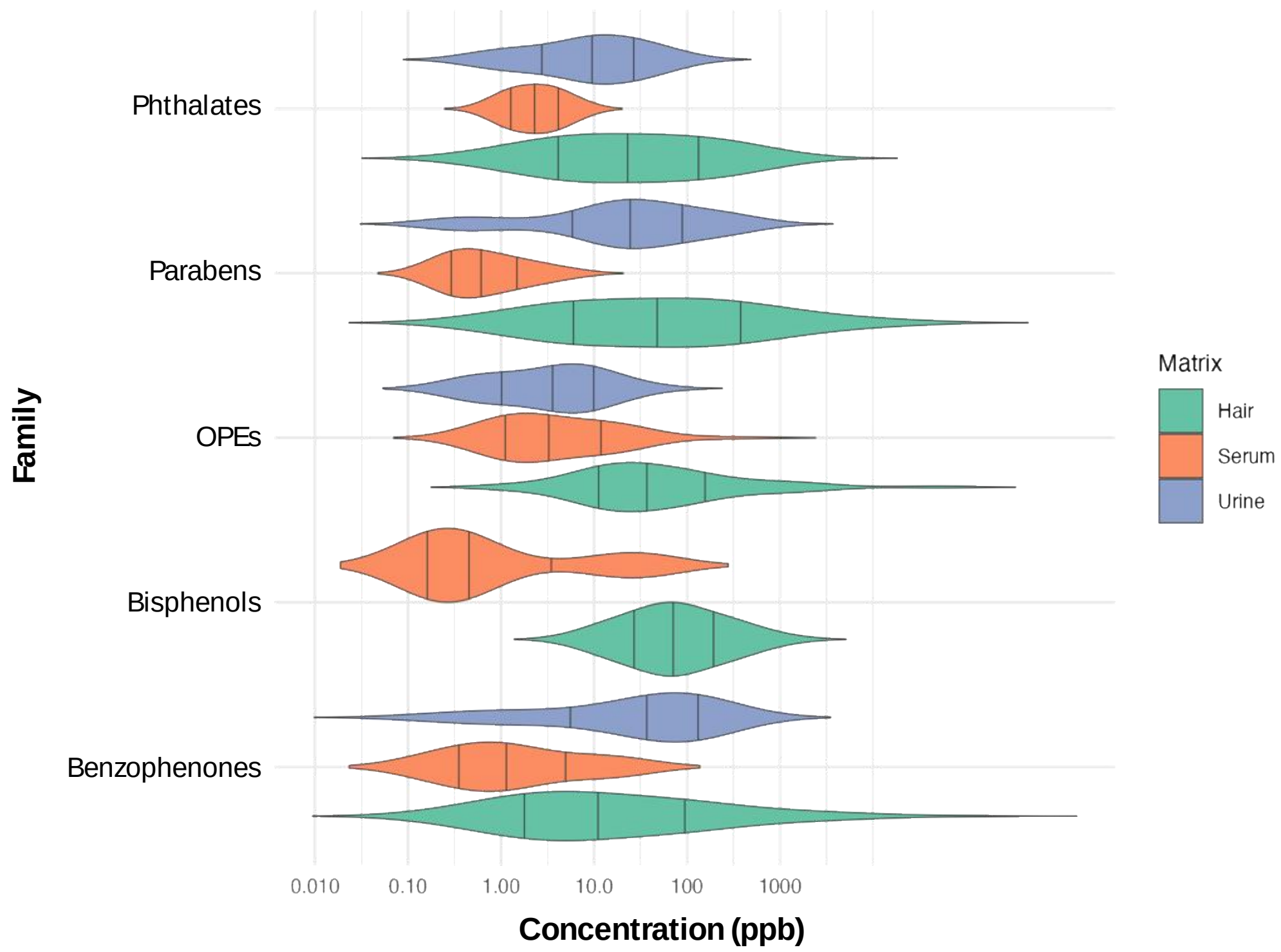

Figure S1. Violin plot of the logarithmic concentration distribution for all chemicals, grouped by chemical families.

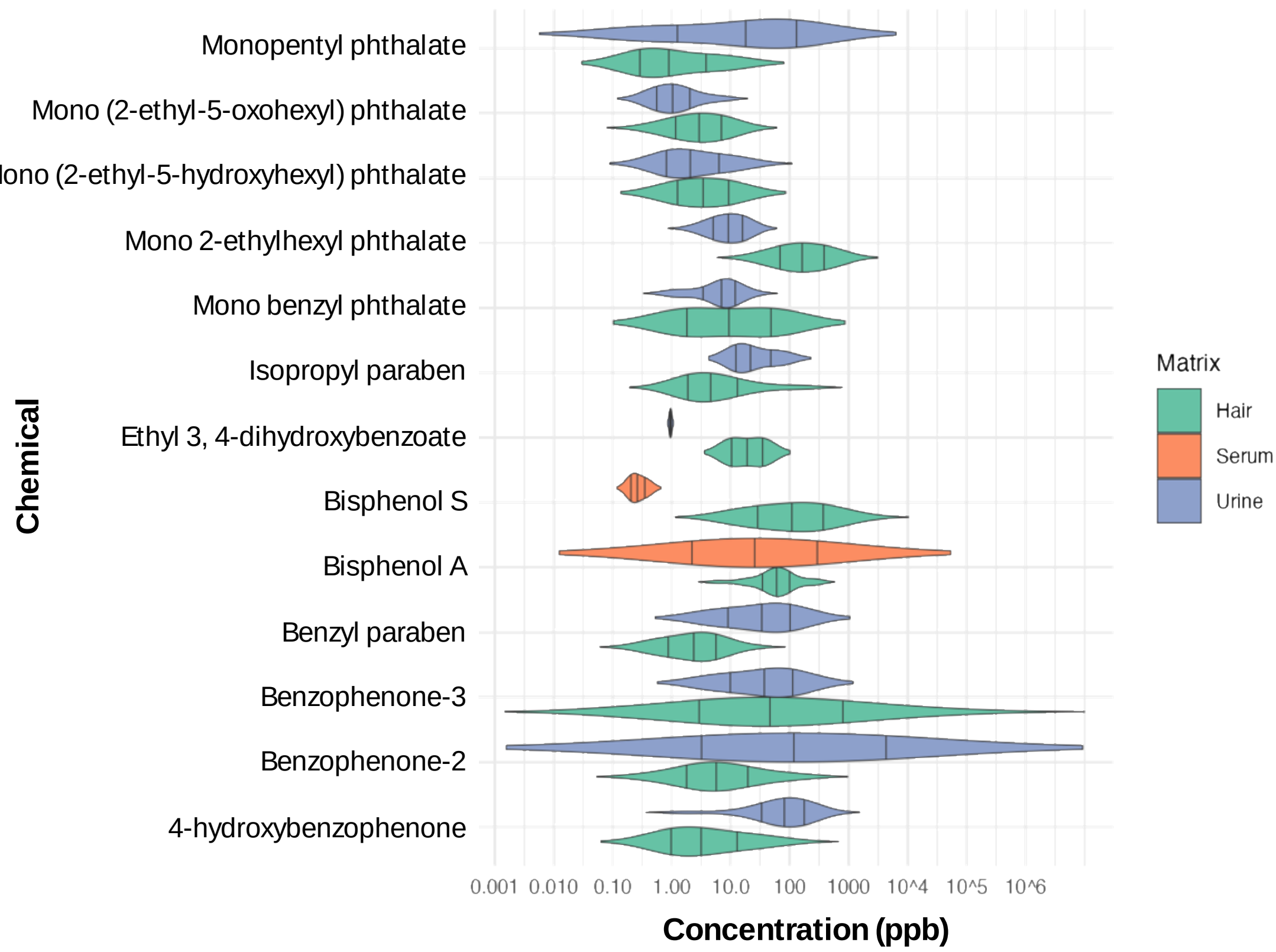

Figure S2. Violin plot of the logarithmic concentration distribution of chemicals found in two matrices (hair-serum, hair-urine, or serum-urine).

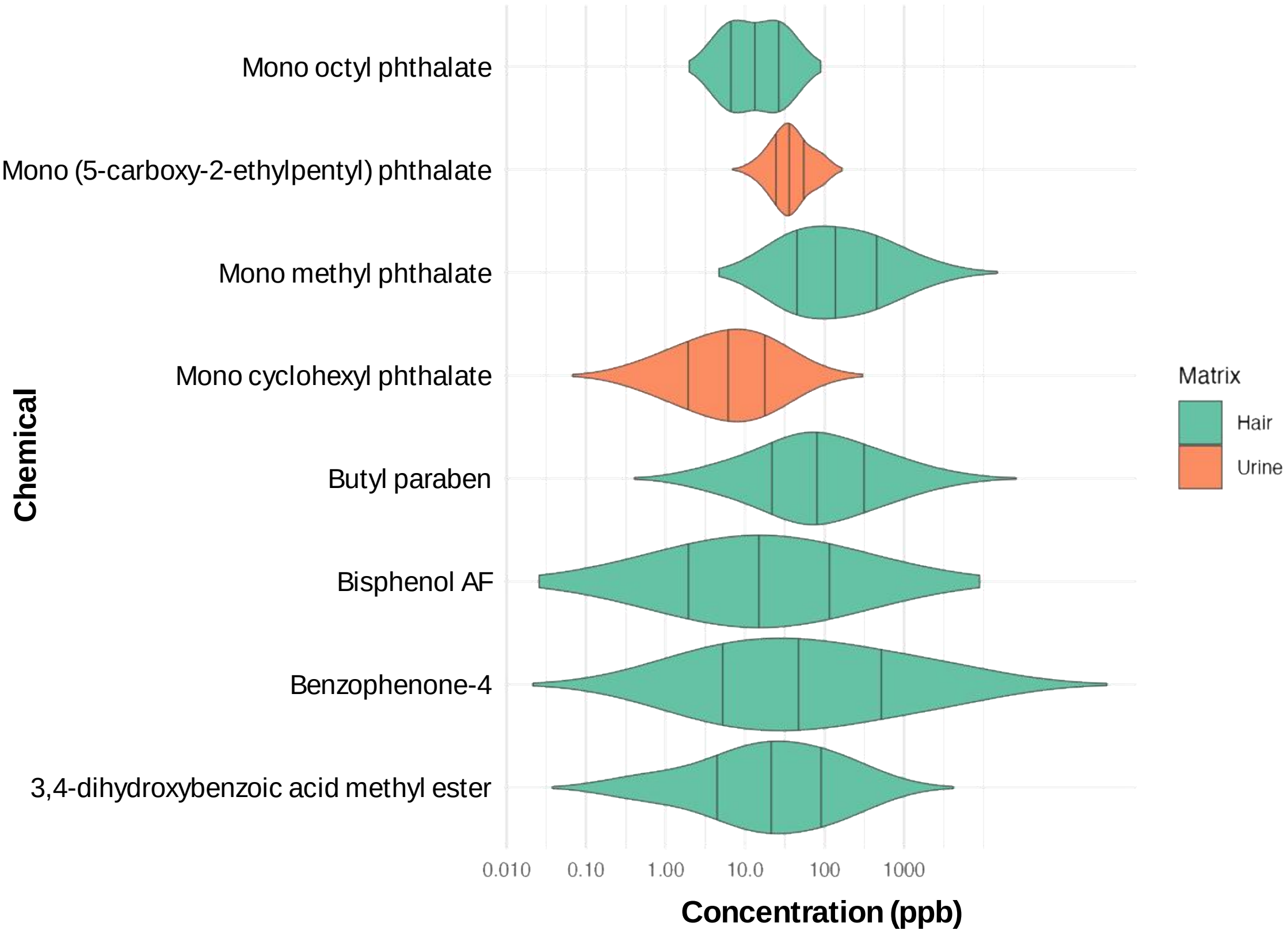

Figure S3. Violin plot of the logarithmic concentration distribution of chemicals detected only in one matrix.

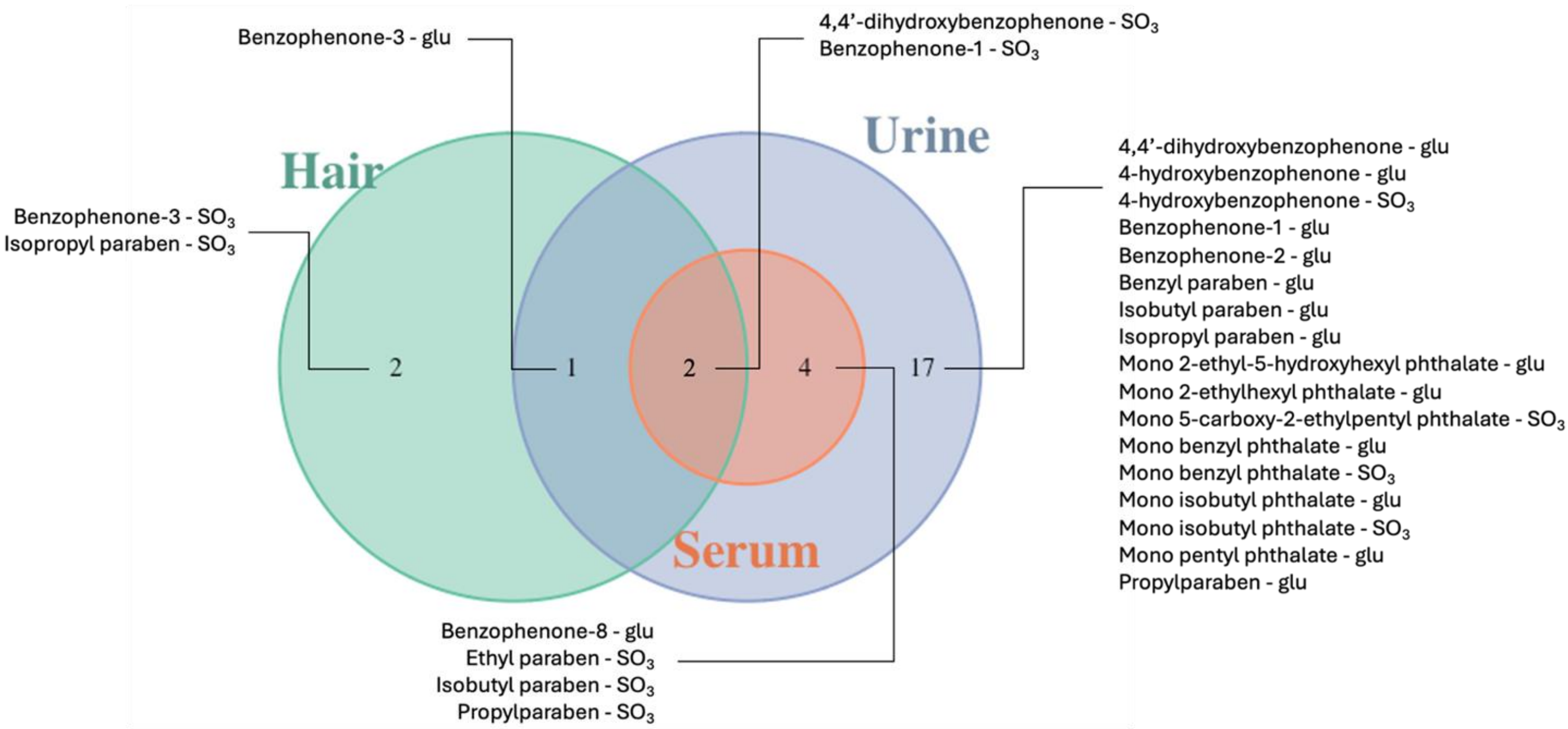

Figure S4. Venn diagram illustrating the number of metabolites detected in each matrix, along with the overlaps representing metabolites common to multiple matrices.

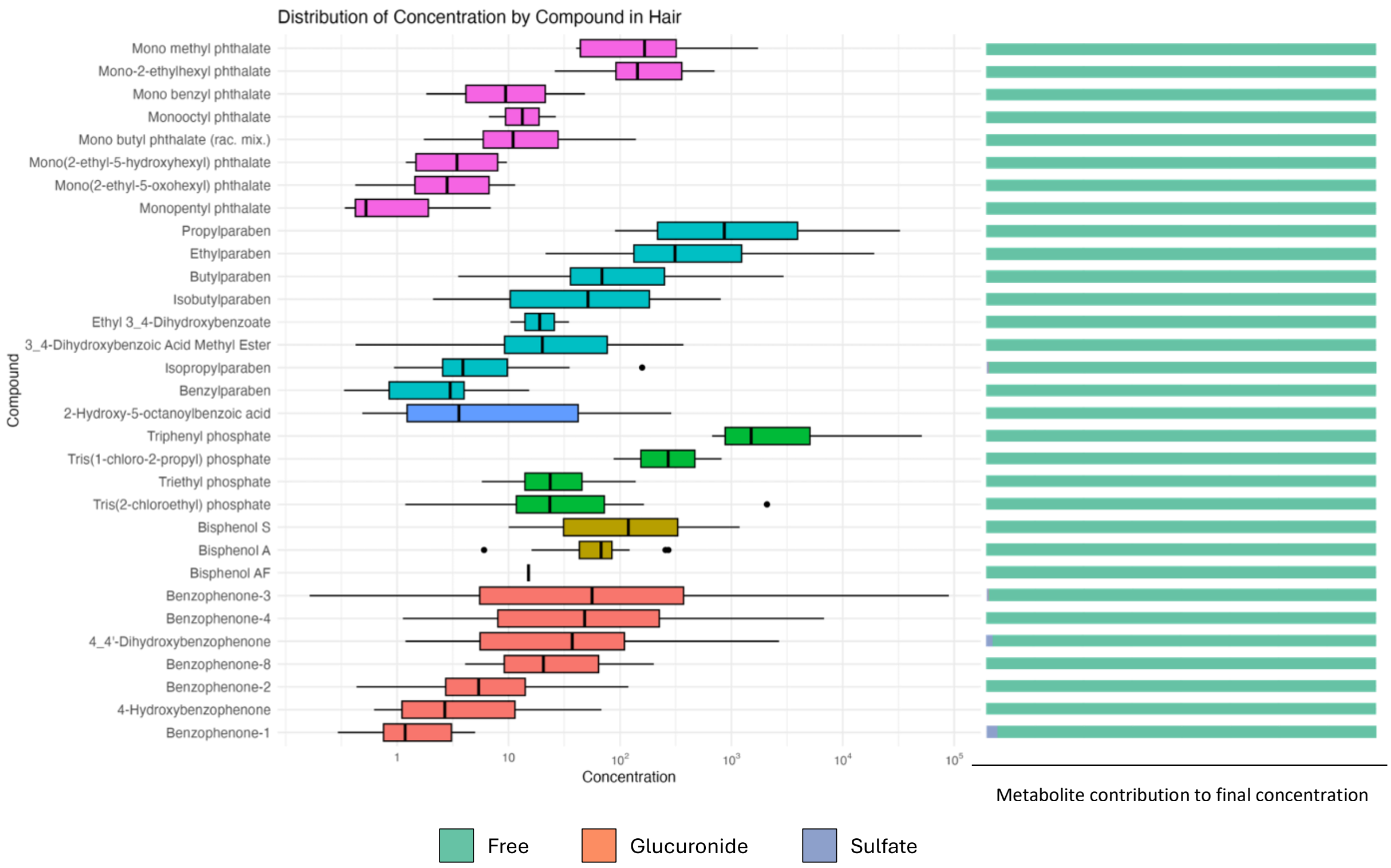
Distribution of Concentration by Compound in Hair
Mono methyl phthalate
Mono-2-ethylhexyl phthalate
Mono benzyl phthalate
Monooctyl phthalate
Mono butyl phthalate (rac. mix.)
Mono(2-ethyl-5-hydroxyhexyl) phthalate
Mono(2-ethyl-5-oxohexyl) phthalate
Monopentyl phthalate
Propylparaben
Ethylparaben
Butylparaben
Isobutylparaben
Ethyl 3_4-Dihydroxybenzoate
3_4-Dihydroxybenzoic Acid Methyl Ester
Isopropylparaben
Benzylparaben
2-Hydroxy-5-octanoylbenzoic acid
Triphenyl phosphate
Tris(1-chloro-2-propyl) phosphate
Triethyl phosphate
Tris(2-chloroethyl) phosphate
Bisphenol S
Bisphenol A
Bisphenol AF
Benzophenone-3
Benzophenone-4
4_4'-Dihydroxybenzophenone
Benzophenone-8
Benzophenone-2
4-Hydroxybenzophenone
Benzophenone-1
Compound
1
10
10^2
10^3
10^4
10^5
Concentration
Metabolite contribution to final concentration
Free
Glucuronide
Sulfate

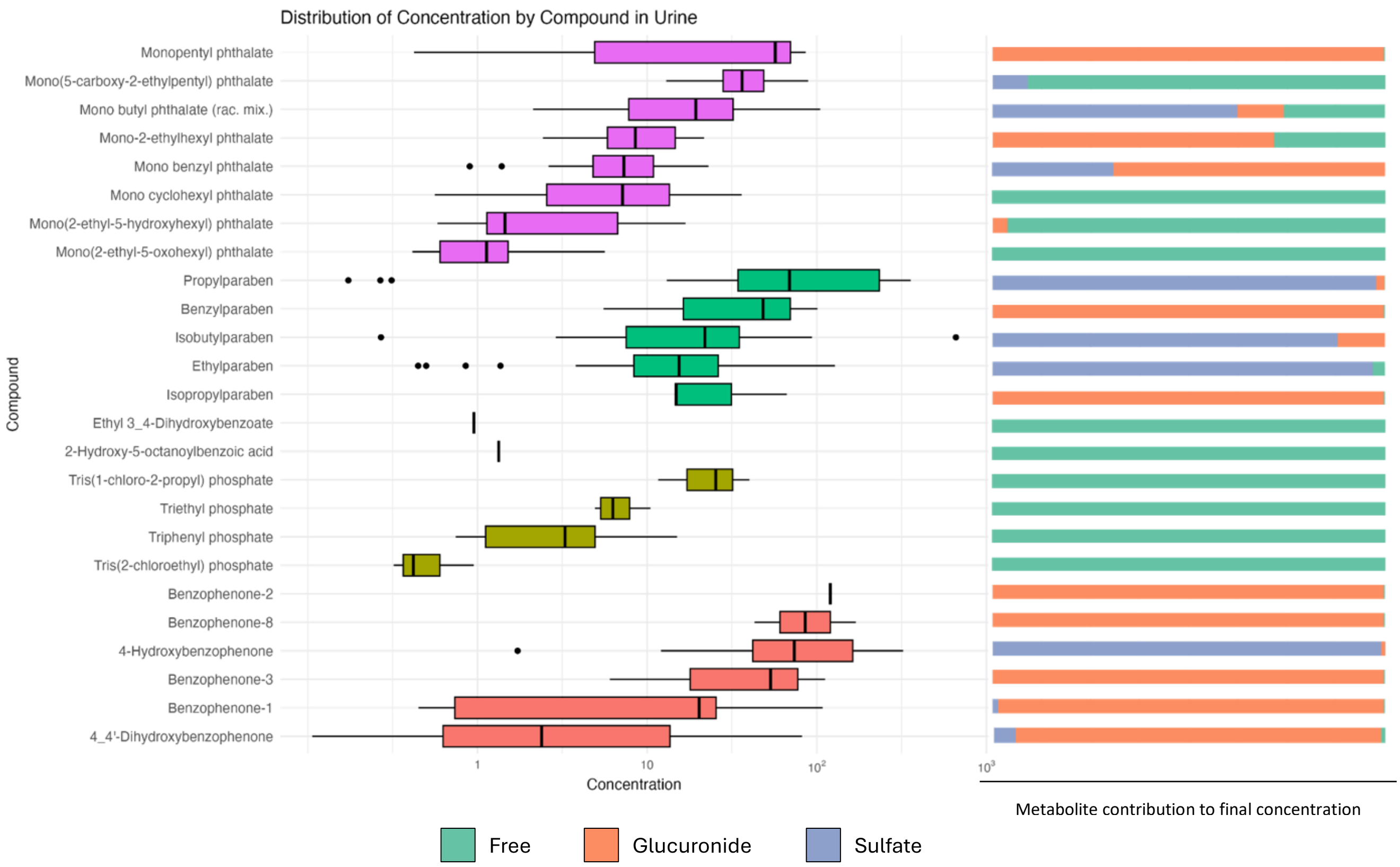
Distribution of Concentration by Compound in Urine
Monopentyl phthalate
Mono(5-carboxy-2-ethylpentyl) phthalate
Mono butyl phthalate (rac. mix.)
Mono-2-ethylhexyl phthalate
Mono benzyl phthalate
Mono cyclohexyl phthalate
Mono(2-ethyl-5-hydroxyhexyl) phthalate
Mono(2-ethyl-5-oxohexyl) phthalate
Propylparaben
Benzylparaben
Isobutylparaben
Ethylparaben
Isopropylparaben
Ethyl 3_4-Dihydroxybenzoate
2-Hydroxy-5-octanoylbenzoic acid
Tris(1-chloro-2-propyl) phosphate
Triethyl phosphate
Triphenyl phosphate
Tris(2-chloroethyl) phosphate
Benzophenone-2
Benzophenone-8
4-Hydroxybenzophenone
Benzophenone-3
Benzophenone-1
4_4'-Dihydroxybenzophenone
Compound
1
10
10^2
10^3
Concentration
Metabolite contribution to final concentration
Free
Glucuronide
Sulfate

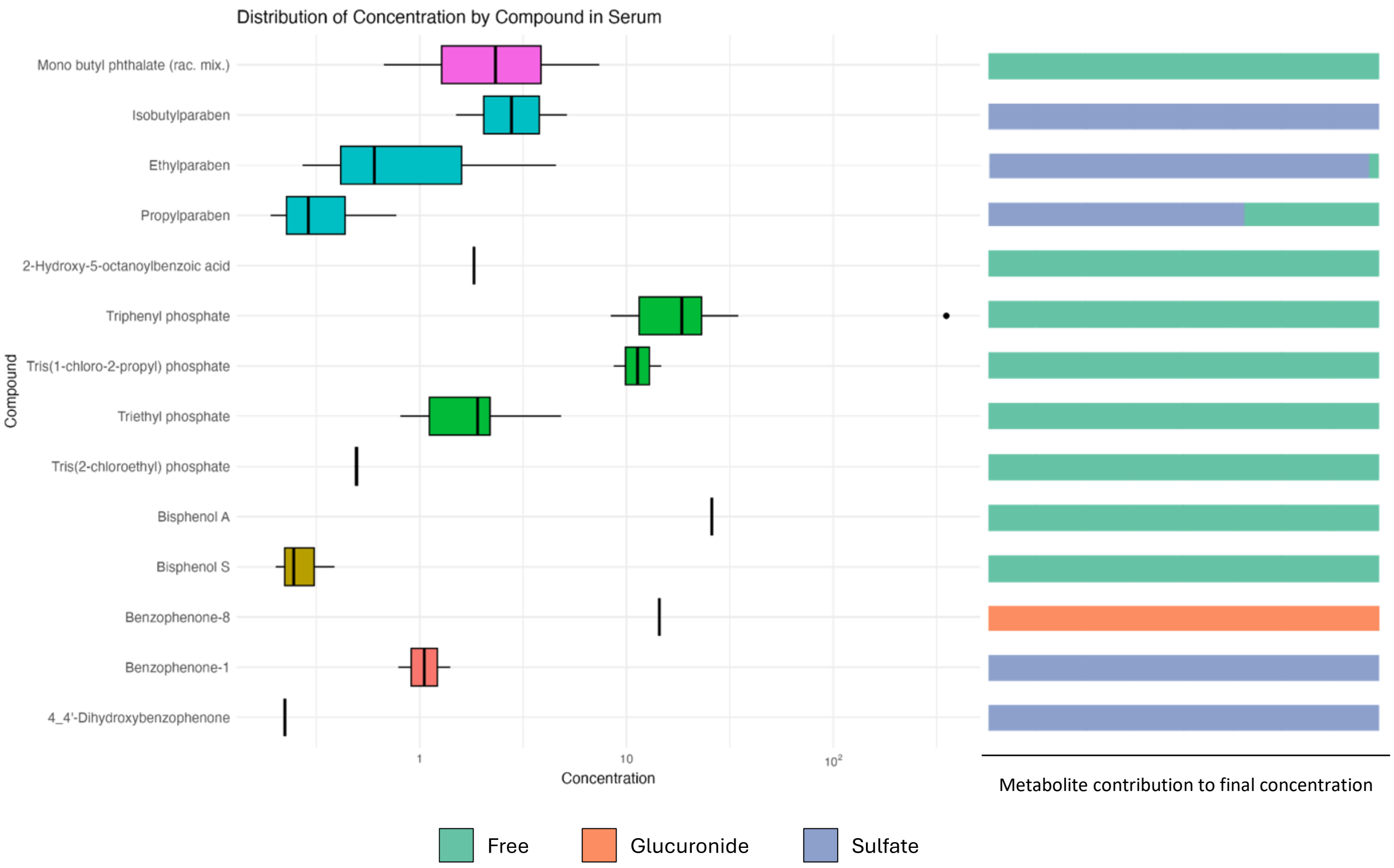

Figure S5. Boxplot showing the distribution of concentrations in each matrix (hair, urine, and serum, in order of appearance) grouped by families and the contribution of each metabolite to the total concentration.

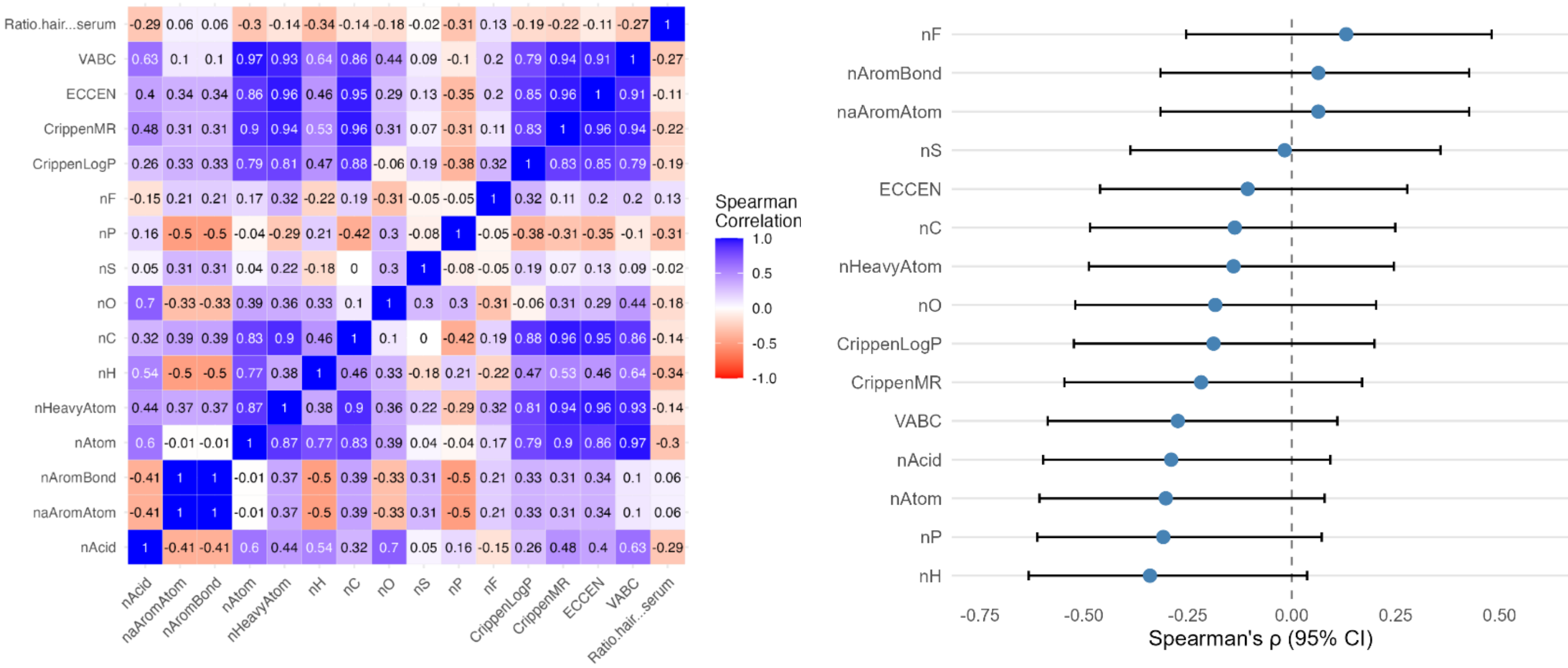

Figure S6. Heatmap of Spearman correlations between the hair-to-serum ratio and physicochemical properties on the left, and forest plot with 95% CI on the right.

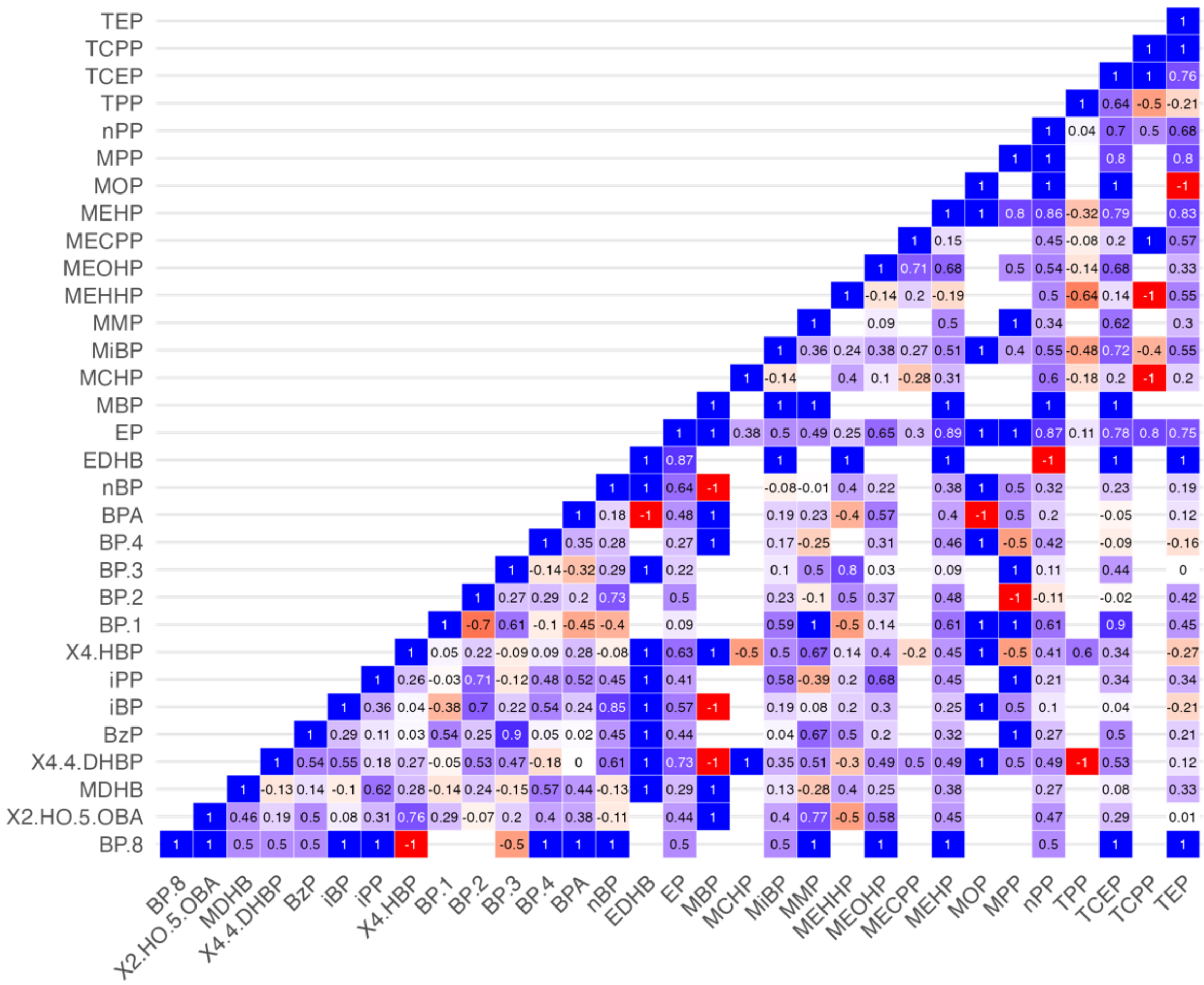

Figure S7. Heatmap of Spearman correlations among chemical compounds.
